# Dissecting Spatiotemporal Structures in Spatial Transcriptomics via Diffusion-Based Adversarial Learning

**DOI:** 10.34133/research.0390

**Published:** 2024-05-29

**Authors:** Haiyun Wang, Jianping Zhao, Qing Nie, Chunhou Zheng, Xiaoqiang Sun

**Affiliations:** ^1^ College of Mathematics and System Sciences, Xinjiang University, Urumqi, China.; ^2^ Department of Mathematics and Department of Developmental and Cell Biology, NSF-Simons Center for Multiscale Cell Fate Research, University of California Irvine, Irvine, CA, USA.; ^3^ School of Artificial Intelligence, Anhui University, Hefei, China.; ^4^ School of Mathematics, Sun Yat-sen University, Guangzhou, China.

## Abstract

Recent advancements in spatial transcriptomics (ST) technologies offer unprecedented opportunities to unveil the spatial heterogeneity of gene expression and cell states within tissues. Despite these capabilities of the ST data, accurately dissecting spatiotemporal structures (e.g., spatial domains, temporal trajectories, and functional interactions) remains challenging. Here, we introduce a computational framework, PearlST (partial differential equation [PDE]-enhanced adversarial graph autoencoder of ST), for accurate inference of spatiotemporal structures from the ST data using PDE-enhanced adversarial graph autoencoder. PearlST employs contrastive learning to extract histological image features, integrates a PDE-based diffusion model to enhance characterization of spatial features at domain boundaries, and learns the latent low-dimensional embeddings via Wasserstein adversarial regularized graph autoencoders. Comparative analyses across multiple ST datasets with varying resolutions demonstrate that PearlST outperforms existing methods in spatial clustering, trajectory inference, and pseudotime analysis. Furthermore, PearlST elucidates functional regulations of the latent features by linking intercellular ligand–receptor interactions to most contributing genes of the low-dimensional embeddings, as illustrated in a human breast cancer dataset. Overall, PearlST proves to be a powerful tool for extracting interpretable latent features and dissecting intricate spatiotemporal structures in ST data across various biological contexts.

## Introduction

Spatial transcriptomics (ST) technologies provides information of the spatial distribution of cells and gene expression [1]. Understanding the relative positioning of transcriptional expression within tissues is crucial for unraveling their spatial architectures and biological functions [2,3]. In recent years, the emerging spatial transcriptome sequencing technologies (e.g., 10X Visium [4], Spatial Transcriptomics [5], Slide-seq [6], Slide-seqV2 [7], HDST [8], seqFISH [9,10], seqFISH+ [11], MERFISH [12,13], and STARmap [14]) have facilitated the exploration of the intricate transcriptional architecture of heterogeneous tissues, prominently advancing our comprehension of the cellular mechanisms underlying various diseases [4,15].

A fundamental challenge in the analysis of ST data lies in the dissection of spatiotemporal structures, encompassing spatial domains, temporal trajectories, and cellular networks. One illustrative instance involves the identification of spatial domains, defined as regions exhibiting coherence in both gene expression and histology, a task analogous to spatial clustering. Traditional clustering methods such as K-means and Louvain methods [16], which rely solely on gene expression, encounter difficulties in producing biologically meaningful outcomes as they overlook spatial and histological information. Methods that employ cell type signatures for deconvolution [17,18] prove unsuitable for achieving cellular or subcellular resolution in ST data.

To address the spatial dependence of gene expression, several methods have been developed. For instance, BayesSpace [19] employs a probability-density [20] approach to clustering by introducing a prior that assigns higher weight to physically close spots. SpecMix [21] enhances the non-negative matrix factorization model of gene expression by integrating graphical models of cellular spatial organization. SEDR [22] utilizes an autoencoder to learn a low-dimensional representation of gene expression, incorporating spatial information through a variational autoencoder. STAGATE [23] employs graph attention networks to learn low-dimensional representations of ST spots. Additionally, some methods incorporate histology images for spatial dependence normalization, such as stLearn [24] and DeepST [25]. SpaGCN [26] integrates spatial location, gene expression, and histological images to construct an undirected weighted graph for spatial clustering.

SpaceFlow [27] initially introduced the concept of the pseudo-spatiotemporal map (pSM) and employed graph convolutional networks to learn latent features of cells or spots in ST data. Building on this, pysodb [28] incorporated the pSM into ST data analysis, utilizing internal functions for implementation. Additionally, stLearn [24] introduced a spatial graph-based method called pseudo-time-space, aiming to model and unveil relationships among dynamic changes in cell transcription states across organizational experiences. Consequently, the construction of spatiotemporal structures has progressively emerged as a key task in the analysis of ST data.

Nevertheless, several challenges persist in effectively characterizing spatiotemporal structures in ST data. Firstly, existing methods face limitations in the effective characterization of spatial features at domain boundaries, often resulting in blurred edges of identified spatial regions. Secondly, the ST expression profile tends to be sparse, primarily due to both technical and biological noises, thereby hindering accurate quantification of expression features and pseudotime estimation. Thirdly, most of the existing methods fall short on deciphering biological functions of the learned latent features within the context of cellular interactions and gene regulations. In addition, tailored models specifically trained on the histology images associated with the ST data are desired to improve the extraction of features from histology images for better spatial analysis.

To address the above gaps, we develop PearlST (partial differential equation [PDE]-enhanced adversarial graph autoencoder of ST) to dissect spatiotemporal structures, including spatial domains, temporal trajectories, and functional interactions of cells or spots, from the ST data. To this end, PearlST learns low-dimensional latent embeddings of ST by leveraging PDE model-based gene expression augmentation and adversarial learning through integrating spatial information, gene expression profiles, and histology image features. We benchmarked PearlST with 10 existing methods and further evaluated its robust performance across multiple ST datasets obtained from various platforms, including 10X Visium, Stereo-seq, Slide-seqV2, MERFISH, and STARmap, and diverse tissues, such as the human dorsolateral prefrontal cortex (DLPFC), mouse visual cortex, mouse olfactory bulb (MOB), mouse embryo, mouse hippocampus, and human breast cancer.

## Results

### Overview of PearlST

**Fig. 1.**
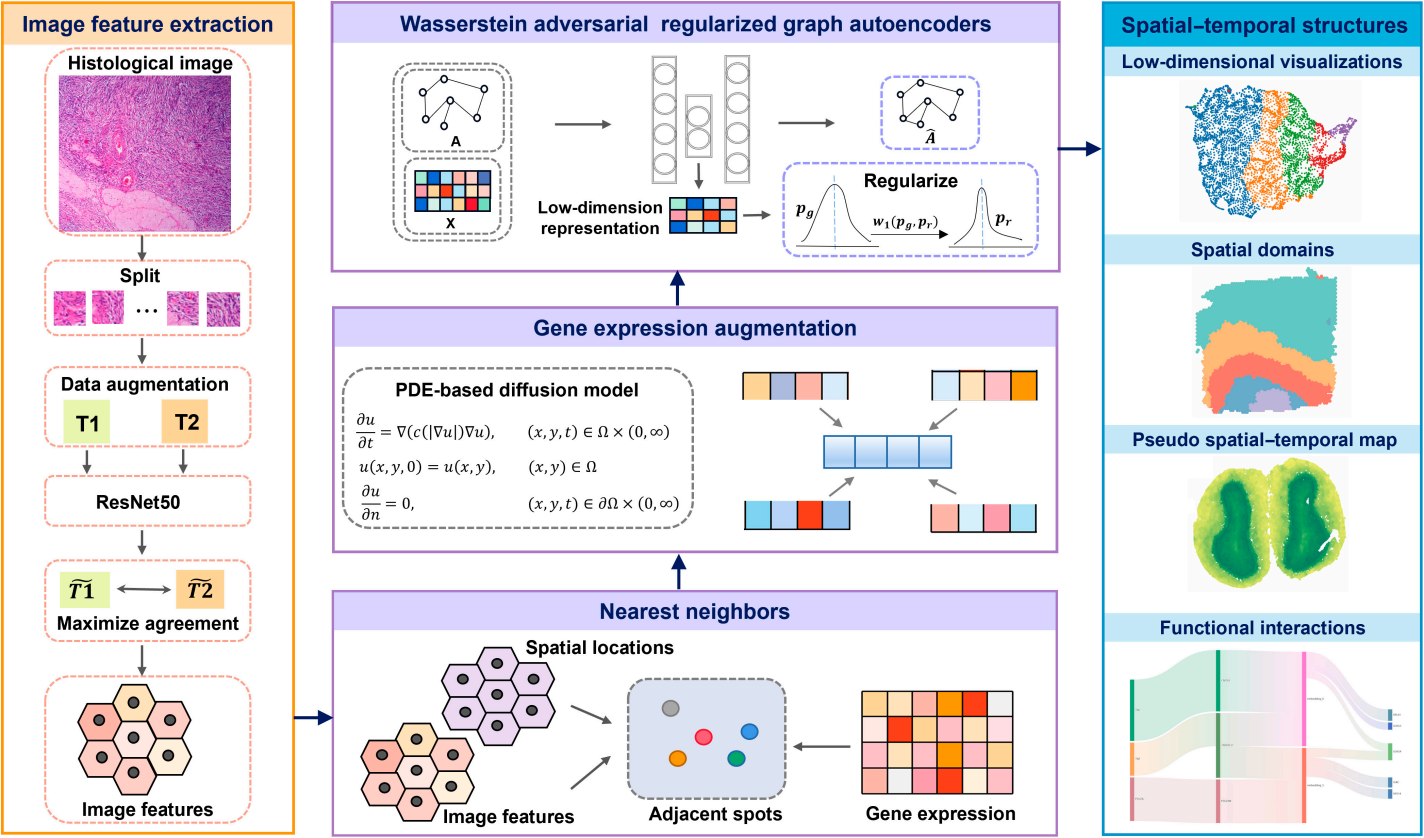
Overview of PearlST. Initially, spatial nearest neighbors are defined for each spot by calculating their similarity using a combination of spatial locations, gene expressions, and histology image features extracted by a tailored SimCLR model. Following this, a PDE-based diffusion model is employed to enhance transcriptional profiling data. Subsequently, a Wasserstein adversarial regularized graph autoencoder is constructed to learn a low-dimensional representation based on the spatial graph and gene expression matrix. Ultimately, the derived low-dimensional representations can be employed for various downstream analyses and applications, including spatial domain identification, trajectory inference, pseudo-spatiotemporal map generation, and analysis of cell–cell communications.

PearlST comprises three integral components: gene expression augmentation, low-dimensional representation learning, and downstream analysis (Fig. 1). First, PearlST enhances gene expression by incorporating information from histology images, spatial location, and gene expression profiles of spatially nearest neighbors for each spot. Notably, PearlST leverages ResNet-50 [29] within the SimCLR framework [30] to learn visual features of each spot image, utilizing histology images collected from diverse tissues across species. To ensure spatial proximity consistency across histology images, spatial locations, and gene expression, the 3 datasets are integrated to identify spatial nearest neighbors for each spot. It is important to emphasize that the feature extraction module is exclusively employed for sequencing platforms that generate histology images, such as 10X Visium. For other ST data, spatial coordinates and gene expression are used to compute the nearest neighbors of the spots. Subsequently, gene expression data undergo denoising and enhancement through a PDE-based diffusion model. Secondly, PearlST learns latent low-dimensional embeddings that encompass both spatial information and augmented gene expressions by employing a Wasserstein adversarial regularized graph autoencoder. The introduction of a Wasserstein regularizer minimizes the 1-Wasserstein distance [31] between distributions. Thirdly, the obtained latent embeddings are utilized for data visualization through Uniform Manifold Approximation and Projection (UMAP) [32]. Based on the latent embeddings, spatial domains are segmented using common clustering algorithms, defaulting to K-means or, alternatively, Louvain clustering. Furthermore, a pSM [27] of the tissue is computed by employing the diffusion pseudotime (DPT) method [33] based on the latent embeddings (Fig. 1).

### Benchmarking PearlST with 10 other methods

**Fig. 2.**
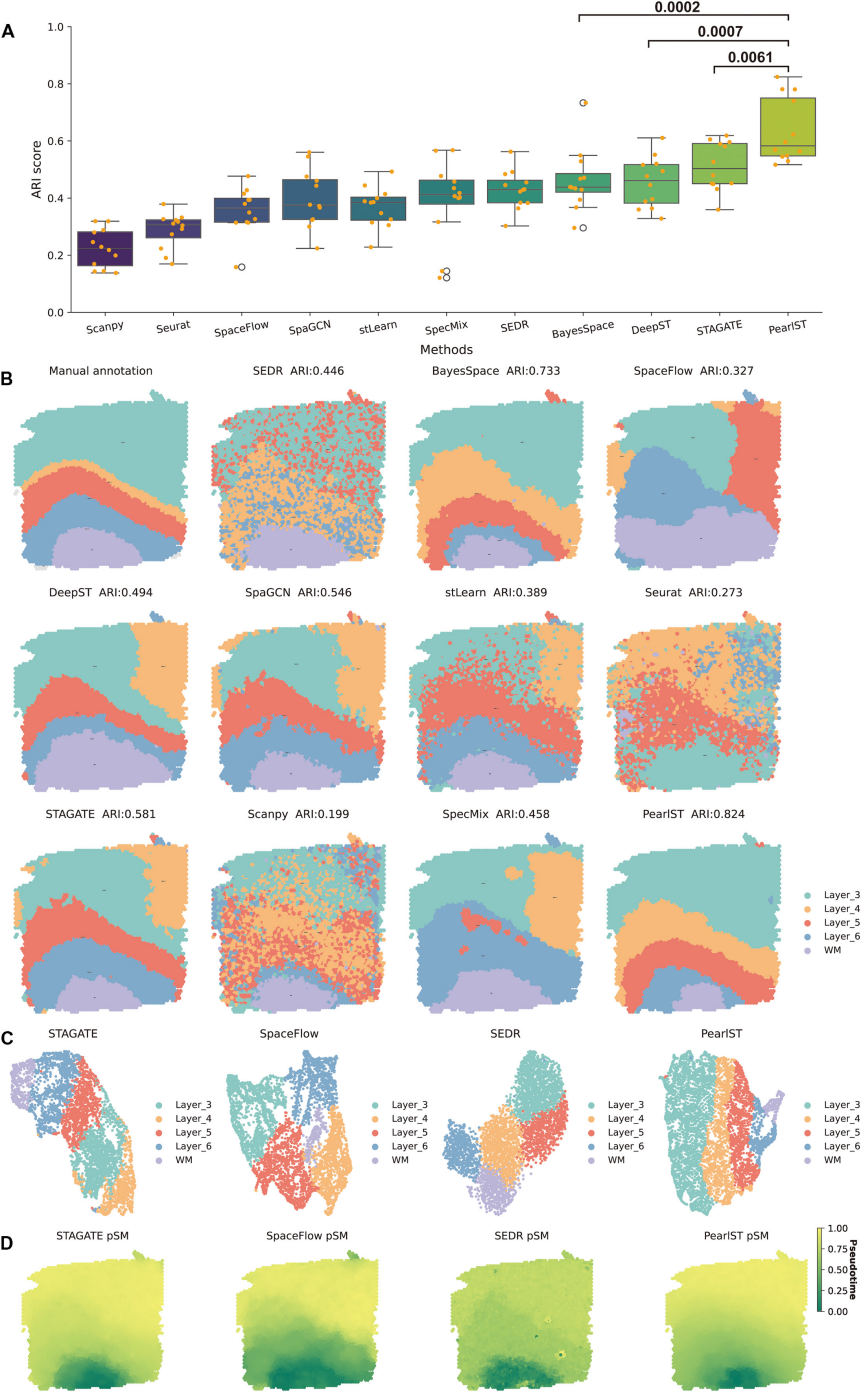
Benchmarking PearlST against other methods in DLPFC tissue analysis. (A) Performance comparison of PearlST with 10 other methods in identifying layer-specific expression patterns across 12 DLPFC samples. (B) Visualization of spatial domain segmentation results obtained by various methods on the 151671 section, with a comparison against manual annotation. (C) UMAP visualization generated by STAGATE, SpaceFlow, SEDR, and PearlST, highlighting the consistency between UMAP results and layer-specific expression profiles. It is important to note that, as end-to-end clustering approaches, the results of SpaGCN and BayesSpace cannot be visualized using UMAP. (D) Pseudo-spatiotemporal map inferred from low-dimensional representations derived from different methods.

To assess the efficacy of PearlST in identifying distinct layer-specific expression profiles, we initially applied it to 12 human DLPFC samples. These samples were previously annotated into 6 cortical layers and white matter (WM) by Maynard et al. [34], considering cytoarchitecture and selected gene markers. Using the manual annotation results as the ground truth, we employed the adjusted Rand index (ARI) to quantify the similarity between the inferred domains and the expert annotations across all 12 sections. In this evaluation, we compared the performance of PearlST with 8 recently proposed spatial clustering approaches (BayesSpace [19], DeepST [25], SpaGCN [26], stLearn [24], SEDR [22], SpaceFlow [27], STAGATE [23], and SpecMix [21]), as well as 2 non-spatial clustering methods implemented by SCANPY [35] and Seurat [36]. We ran each method according to the official technical documentation and used the default parameters for each method (Note S1). The comparison results reveal that PearlST achieves a significantly higher median ARI score than the other methods across the 12 samples (Fig. 2A). Following that, STAGATE, DeepST, and BayesSpace have higher median ARI scores on these datasets. We conducted a one-sided Wilcoxon rank-sum test comparing the results of PearlST with these 3 methods. The statistical test results indicate that PearlST performs significantly better in clustering on the DLPFC dataset compared to STAGATE (*P* = 6.1 × 10^−3^), DeepST (*P* = 7 × 10^−4^), and BayesSpace (*P* = 2 × 10^−4^). Notably, SEDR and SpecMix exhibit comparable clustering performance.

Subsequently, we conducted a more detailed analysis on section 151671 (Fig. 2B to D). We visualized the domain segmentation results obtained by each method and compared them to expert annotations (Fig. 2B). Notably, all methods exhibited challenges in capturing the subtle structure of Layer 4, potentially attributed to the spatial resolution limitations inherent in this specific ST data. Despite this, both PearlST and BayesSpace successfully captured the remaining structures (Layer 3, Layer 5, Layer 6, and WM) as observed in the manual annotations. Furthermore, it was observed that the expression profiles of specific layers segmented by BayesSpace had more pronounced jagged edges, while the results from PearlST appeared relatively smoother. Specifically, for the subtle Layer 4 structure, BayesSpace annotated it as a large area, whereas PearlST provided a smaller and more refined annotation result. Other methods also seemed to dissect section 151671 into 5 layers, but there was more noise at the edges of layer-specific expression profiles, particularly for SCANPY and Seurat. Similar comparison results were observed across the remaining 11 samples (Figs. S1 to S3).

Additionally, we illustrate that PearlST excels in revealing distances between spatial domains and depicting spatial trajectories in UMAP plots. To achieve this, we utilized SCANPY to reduce the low-dimensional representation of the output from various methods to 2 dimensions for UMAP visualization and PAGA (partition-based graph abstraction) trajectory inference. Notably, the UMAP plots generated by embeddings from PearlST and STAGATE exhibited well-organized cortical layers and consistent spatial trajectories, progressing from WM to Layer 3 (Fig. 2C and Fig. S4). Among the other methods, only DeepST and SpecMix results demonstrated somewhat similar consistency to PearlST. In the PAGA graphs (Fig. S5), the low-dimensional representations of PearlST displayed an approximately linear developmental trajectory from Layer 3 to Layer 6, with stronger similarity observed between adjacent layers compared to non-adjacent layers. Although the low-dimensional representation of DeepST also showcased a linear trajectory, it did not correctly depict the progression from Layer 3 to Layer 6.

In the final analysis, we compared the pSM inferred by different methods, specifically focusing on spatially aware methodologies. STAGATE, SpaceFlow, SEDR, and PearlST all presented layer-patterned pSMs with a distinct and smooth color gradient (Fig. 2D). Notably, these maps suggested a pseudo-spatiotemporal ordering from WM to Layer 3, aligning with the developmental order of the cortex from the inside out and reflecting the hierarchical spatial organization of the tissue. However, it is crucial to highlight that only the results from PearlST exhibited a clear and unique developmental starting point, along with a smooth progression between neighboring layers (Fig. 2D and Fig. S6).

### PearlST augments gene expression for better characterizing spatial expression patterns

Subsequently, we illustrate the efficacy of the PDE-based diffusion model in noise reduction for ST data, emphasizing its ability to characterize spatial expression patterns. PearlST was applied to the 12 DLPFC samples utilized in the previous section, and ablation experiments were designed to investigate the impact of data enhancement on downstream analyses. The findings revealed that enhancing gene expression data significantly improved the accuracy of spatial domain identification (Fig. S7). For further validation, we selected 5 of these samples to demonstrate that the augmented data facilitated other spatiotemporal structure inference tasks as well (Fig. S8). In the low-dimensional visualization results, the enhanced data showcased a clear hierarchical structure in samples 151507, 151670, 151672, and 151675, while the results from the raw data were accompanied by a substantial amount of noise, and the original hierarchical structures were not even discernible in samples 151507 and 151672 (Fig. S8A). The enhanced gene expression data notably contributed to the generation of more consistent PAGA trajectory inferences, particularly evident in samples 151670 and 151672 (Fig. S8B). On these few samples, neither the raw nor the augmented data produced pSMs that exhibited a high degree of agreement with the true developmental order; however, the latter results demonstrated a clearer and more progressive order (Fig. S8C).

### PearlST depicts the developmental trajectory in STARmap data of mouse visual cortex tissue

**Fig. 3.**
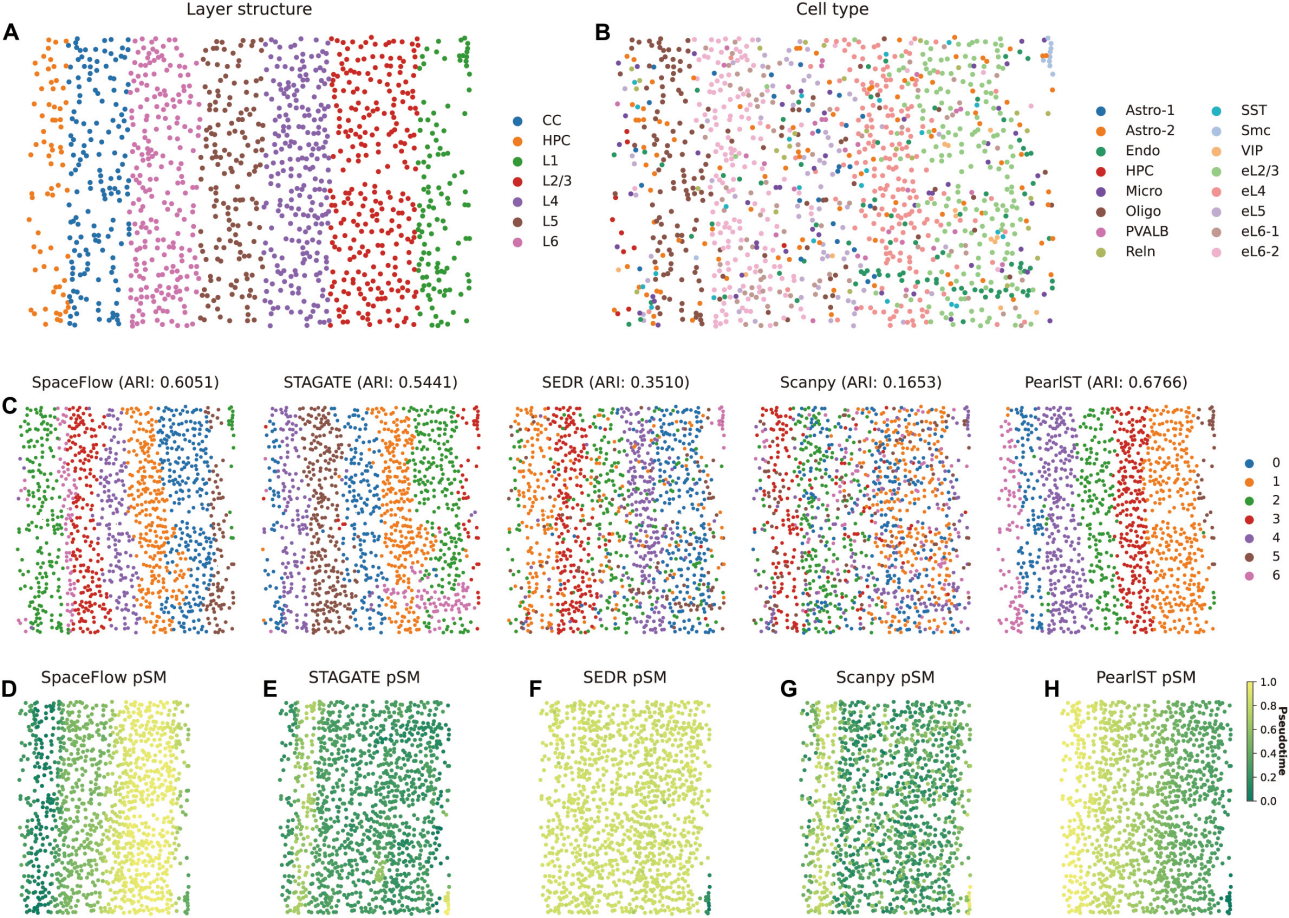
PearlST depicts the developmental trajectory in STARmap data of mouse visual cortex tissue. (A) Expert-annotated structural layers within the mouse visual cortex. (B) Visualization depicting the distribution of each cell type within the ST dataset. (C) Comparative performance assessment of SpaceFlow, STAGATE, SEDR, SCANPY, and PearlST in identifying spatial domains on the mouse visual cortex dataset. (D to H) Visualization of pseudo-spatiotemporal maps (pSMs) calculated using low-dimensional embeddings generated by SpaceFlow (D), STAGATE (E), SEDR (F), SCANPY (G), and PearlST (H).

Furthermore, we applied PearlST to an image-based ST dataset generated by STARmap [14] with single-cell resolution. The STARmap dataset comprises 1,020 genes expressed on 1,207 cells and has been annotated with 7 structural layers (Fig. 3A) and 16 cell types (Fig. 3B). Similar to previous analyses, we initially clustered the ST data using PearlST, considering expert annotations as the gold standard, and compared the results with 4 other methods. The number of clusters for all compared approaches was set to 6. PearlST demonstrated the most accurate domain segmentation with an ARI score of 0.67. SpaceFlow followed with an ARI of 0.60 and STAGATE was 0.54, while the remaining 3 methods yielded clustering results with ARI scores below 0.4 (Fig. 3C). These results are further manifested in the distribution of specific regions, revealing more overlap between regions produced by SCANPY and SEDR. In contrast, SpaceFlow, STAGATE, and PearlST exhibited significantly less overlap. While SpaceFlow showed slight overlap on CC and HPC and larger overlap on HPC and L6, wrongly identifying a portion of L1 as L2/3, PearlST only annotated a small portion of L1 to L2/3 and very few to L5 (Fig. 3C). The outputted low-dimensional representations from these methods were used to compute the pSM. SCANPY exhibited the least satisfactory result, lacking a clear differentiation trajectory (Fig. 3G). Both STAGATE and SEDR results did not show a gradient process, with dark green dominating the former and light green dominating the latter (Fig. 3E and F). SpaceFlow demonstrated a satisfactory progression from the left to the center; however, it presented an anomalous jump from the very first developmental stage on the right to the final form in the center (Fig. 3D). In contrast, the results from PearlST were the most satisfying, displaying a gradual color change from darker to lighter from left to right, effectively unveiling the developmental trajectory of the mouse visual cortex (Fig. 3H).

### PearlST uncovers laminar structures in Stereo-seq data of coronal MOB tissue

**Fig. 4.**
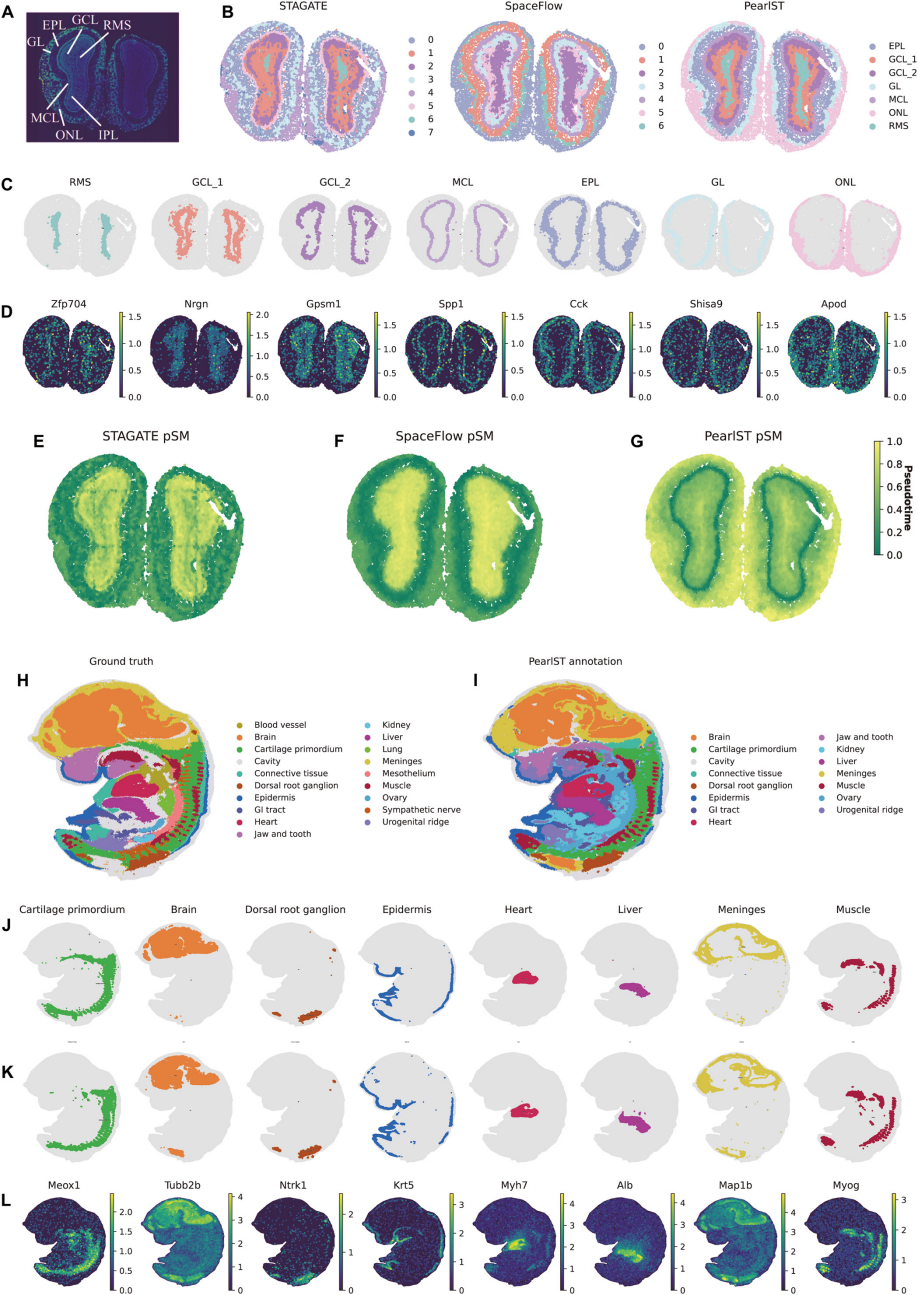
PearlST identifies laminar organization in Stereo-seq data of the mouse olfactory bulb (MOB) tissue and reveals finer-grained tissue structures in Stereo-seq data of mouse embryo. (A) Laminar organization of MOB annotated in the DAPI-stained image generated by Stereo-seq. (B) Spatial domains segmented by STAGATE, SpaceFlow, and PearlST on the Stereo-seq data of the MOB tissue. (C) Visualization of each domain identified by PearlST on the MOB tissue dataset. (D) Visualization of marker genes for each domain annotated by PearlST on the MOB tissue. (E to G) pSMs generated by SCANPY on the low-dimensional embedding of STAGATE (E), SpaceFlow (F), and PearlST (G). (H) Mouse embryo data at E12.5 annotated by Chen et al., treated as ground truth. (I) The annotation results of PearlST on the mouse embryo dataset, comprising 16 regions, with 3 regions unannotated compared to the ground truth. (J) Visualization of each region annotated by Chen et al. on mouse embryo data. (K) Visualization of each region identified by PearlST on mouse embryo data. (L) Visualization of marker genes for each region identified by PearlST.

We demonstrate here that PearlST excels in delineating the laminar structure of data acquired with Stereo-seq [37]. Stereo-seq is an emerging spatial omics technology that provides subcellular spatial resolution through DNA nanopore pattern array chips. For validation, we utilized a coronal MOB tissue dataset, a widely used model tissue with laminar organization. Fu et al. [22] annotated the laminar organization in the DAPI-stained image, containing the rostral migratory stream (RMS), granule cell layer (GCL), internal plexiform layer (IPL), mitral cell layer (MCL), external plexiform layer (EPL), olfactory nerve layer (ONL), and glomerular layer (GL) (Fig. 4A). SpaceFlow and PearlST partitioned this dataset into 7 layers, and STAGATE annotated the dataset into 8 layers according to original tutorial (Fig. 4B). Both PearlST and STAGATE provided well-defined layer structures and effectively demarcated the GL and MCL regions, while SpaceFlow results appeared to have extensive overlap. Subsequently, we applied marker genes from each anatomical region to validate PearlST results (Fig. 4C and D). PearlST demonstrated good correspondence between clusters and known marker genes. Some marker genes, such as Mbp and Pcp4, showed high expression levels overlapping with neighboring regions, which is expected due to shared cell types in different internal structures of organs. Overall, PearlST accurately identified relevant anatomical regions. Finally, we computed the pSM using the low-dimensional representations of each method. The pSM values (green) were lowest in the EPL and increase as they move toward the interior. This sequence of pSM coincides with the developmental order of these layers, starting in the central EPL and developing outward toward the sides, leading to the MCL and the GL, ONL, and GCL developing last [38]. Values are highest in the interior, the peaks being in the GCL and RMS. This suggests that spatial flow calculations of pSM are not only clearer than non-spatial pseudotime estimation but also more accurately reflect the temporal and developmental relationships between cells. Overall, the pSM calculated by PearlST (Fig. 4G) is in high agreement with the real developmental order in these layers. In contrast, the pSM results from both STAGATE (Fig. 4E) and SpaceFlow (Fig. 4F) indicate a developmental direction from the outermost IPL layer to the middle-most MCL layer, which is inconsistent with the true developmental order.

### PearlST reveals finer-grained tissue structures in Stereo-seq data of mouse embryo

In this example, we demonstrate that PearlST excels in revealing finer-grained tissue structures using Stereo-seq data of a mouse embryo at E12.5, containing 55,295 bins and 27,330 genes [37]. Chen et al. [37] annotated the mouse embryo E12.5 transcriptional profile into 19 domains (Fig. 4H). Initially, we performed spatial clustering of this dataset using PearlST, setting the number of clusters to 19. The clustering results by PearlST matched the annotated regions well (Fig. 4I). PearlST effectively captured most of the fine-grained structures in the embryo, demonstrating high consistency with the original annotations. Major regions such as the cartilage primordium, brain, heart, dorsal root ganglion, liver, and muscle regions were accurately identified (Fig. 4I). However, there were 3 regions (blood vessel, lung, and sympathetic nerve) that PearlST did not annotate accurately. Further visualization and comparison of annotations by PearlST and the ground truth for each major region demonstrated higher consistency between them (Fig. 4J and K). Importantly, PearlST annotations exhibited high concordance with known marker genes of major organs (Fig. 4L). For instance, the cartilage primordium region is marked by Meox1, Pax1, and Pax9; the dorsal root ganglion region is marked by Prdm12 and Ntrk1; epidermis is marked by Krt5, Krt14, and Krt15; heart is marked by Nppa, Myl7, Myl2, and Myh7; liver is marked by Afp, Alb, and Fgb (Fig. 4L and Figs. S10 and S11). Additionally, there are regions marked by individual genes, such as the brain region marked by Tubb2b and the meninges region marked by Map1b.

### PearlST accurately dissects the relevant anatomical regions in the Slide-seqV2 data of mouse hippocampus and MOB tissues

**Fig. 5.**
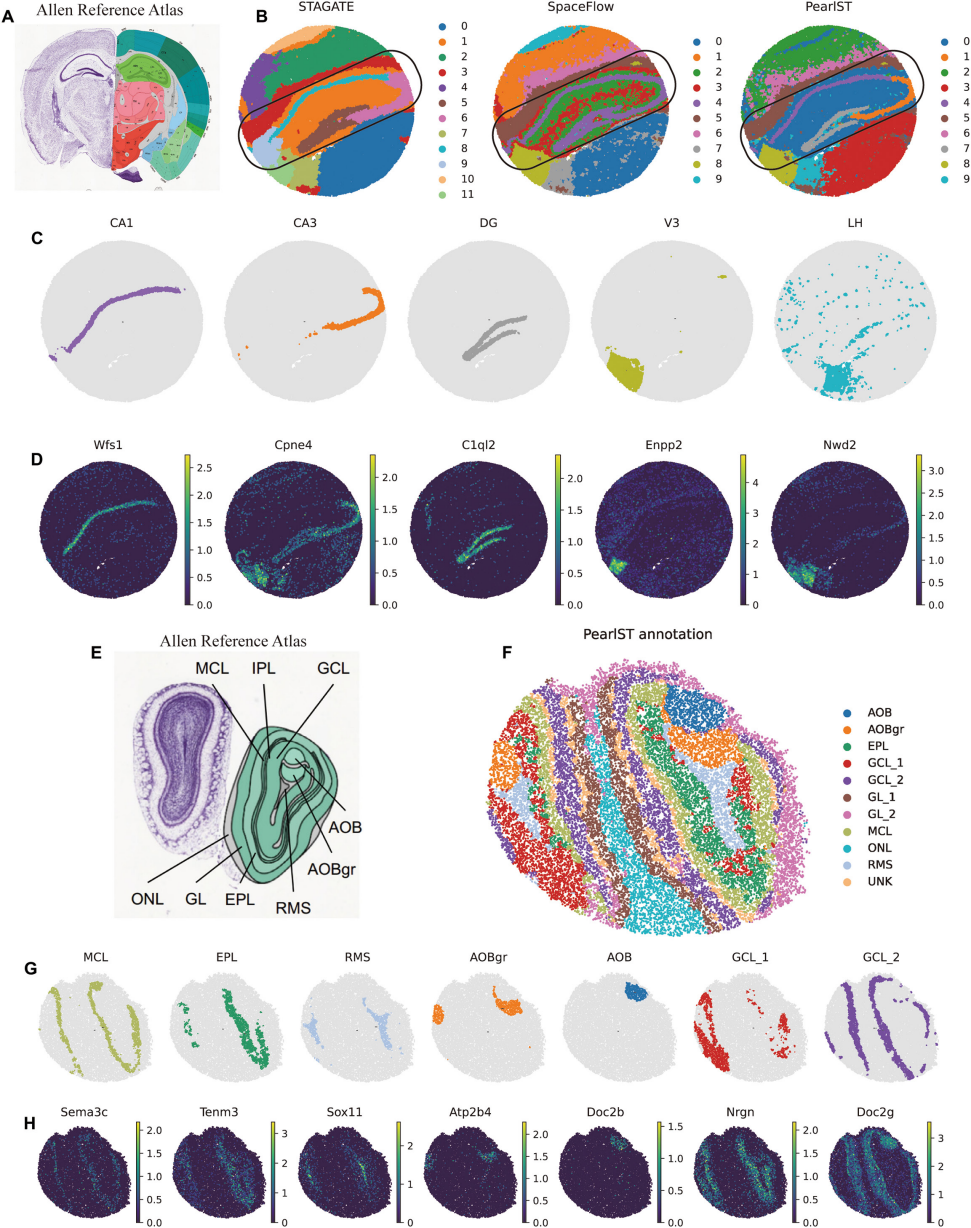
PearlST accurately dissects the relevant anatomical regions in Slide-seqV2 datasets of mouse hippocampus and mouse olfactory bulb tissues. (A) Annotation results for mouse hippocampus tissue based on the Allen Reference Atlas. (B) Clustering results of STAGATE, SpaceFlow, and PearlST for the mouse hippocampus tissue dataset, with the number of clusters set to 9 classes. (C) Visualization of each region in mouse hippocampal tissue under PearlST annotation results. (D) Visualization of marker gene expression corresponding to each region in mouse hippocampal tissue. (E) Annotation of MOB tissue based on the Allen Reference Atlas. (F) Visualization of PearlST annotation results on the MOB tissue dataset. (G) Visualization of each region identified by PearlST on the MOB data. (H) Visualization of marker genes for each domain identified by PearlST on the MOB tissue. PearlST reveals microenvironment heterogeneity and functional cellular interactions in 10X Visium data of human breast cancer.

We further employed Slide-seqV2 ST data with a spatial resolution of 10 μm to evaluate the performance of PearlST. Initially, we applied PearlST to a Slide-seqV2 dataset from the mouse hippocampus [7], annotated by the Allen Reference Atlas [39] (Fig. 5A). As anticipated, PearlST effectively characterized the tissue structure, revealing distinct spatial domains. In comparison, the clusters identified by SpaceFlow lacked significant spatial separation (Fig. 5B). Notably, PearlST and STAGATE depicted a clear “rope-like” structure and an “arrowhead-like” structure in the hippocampal region, accurately identifying its 5 spatial domains. This result is consistent with the annotation of hippocampal structures in the Allen Reference Atlas (Fig. 5B). All 3 methods used for comparison produced spatially consistent clustering, capturing major anatomical regions such as the dentate gyrus (DG) and the pyramidal layers within Ammon’s horn, further divided into CA1, CA2, and CA3 regions. In particular, PearlST and STAGATE exhibited sharper boundaries in delineating the CA3 and DG regions, aligning more closely with anatomical annotations (Fig. 5A and C) and marker gene expression (Fig. 5D). In contrast, SpaceFlow annotated CA1, CA3, and DG as the same region. Additionally, both PearlST and SpaceFlow annotated the LH and V3 regions. To validate the clustering performance of PearlST, we examined the marker gene expression of the regions (Fig. 5D), revealing good agreement between most regions and their corresponding marker genes. This further supports the robustness and accuracy of PearlST in ST data analysis.

Furthermore, we applied PearlST to sections of the MOB sequenced by Slide-seqV2 [7] and observed that the spatial domains identified by PearlST were consistent with the annotations in the Allen Reference Atlas (Fig. 5E). As previously mentioned, Fu et al. [22] annotated the laminar organization of the coronal MOB in the DAPI-stained image, including the RMS, GCL, IPL, MCL, EPL, ONL, and GL. In this case, PearlST annotated the dataset into 10 regions, as in a previous study [23]. Specifically, PearlST identified 2 spatial domains corresponding to the accessory olfactory bulb (AOB) and the granular layer of the accessory olfactory bulb (AOBgr), respectively (Fig. 5F).

The spatial domains uncovered by PearlST are strongly supported by known gene markers (Fig. 5G and H). For instance, the gene Nrgn exhibited high expression in the GCL_1 domain, while the gene Gap43 showed strong expression in the segmented region AOB, consistent with immunohistochemical experimental results [40]. The granular cell marker Atp2b4 [41] displayed strong expression in the identified AOBgr domain, and the mitral cell marker Gabra1 [42] exhibited dominant expression in the narrow MCL structure, also identified by PearlST. Notably, PearlST identified a spatial subgroup called GCL_1 that is predominantly expressed by Nrgn. Considering that Nrgn is a well-documented risk gene for schizophrenia [43], this suggests a potential association between this structural domain and cognitive function. In summary, these results demonstrate the capability of PearlST to identify spatial domains in Slide-seqV2 sequencing data with much higher spatial resolution, enabling the exploration of tissue structures in finer detail.

### PearlST reveals microenvironment heterogeneity and functional cellular interactions in 10X Visium data of human breast cancer

**Fig. 6.**
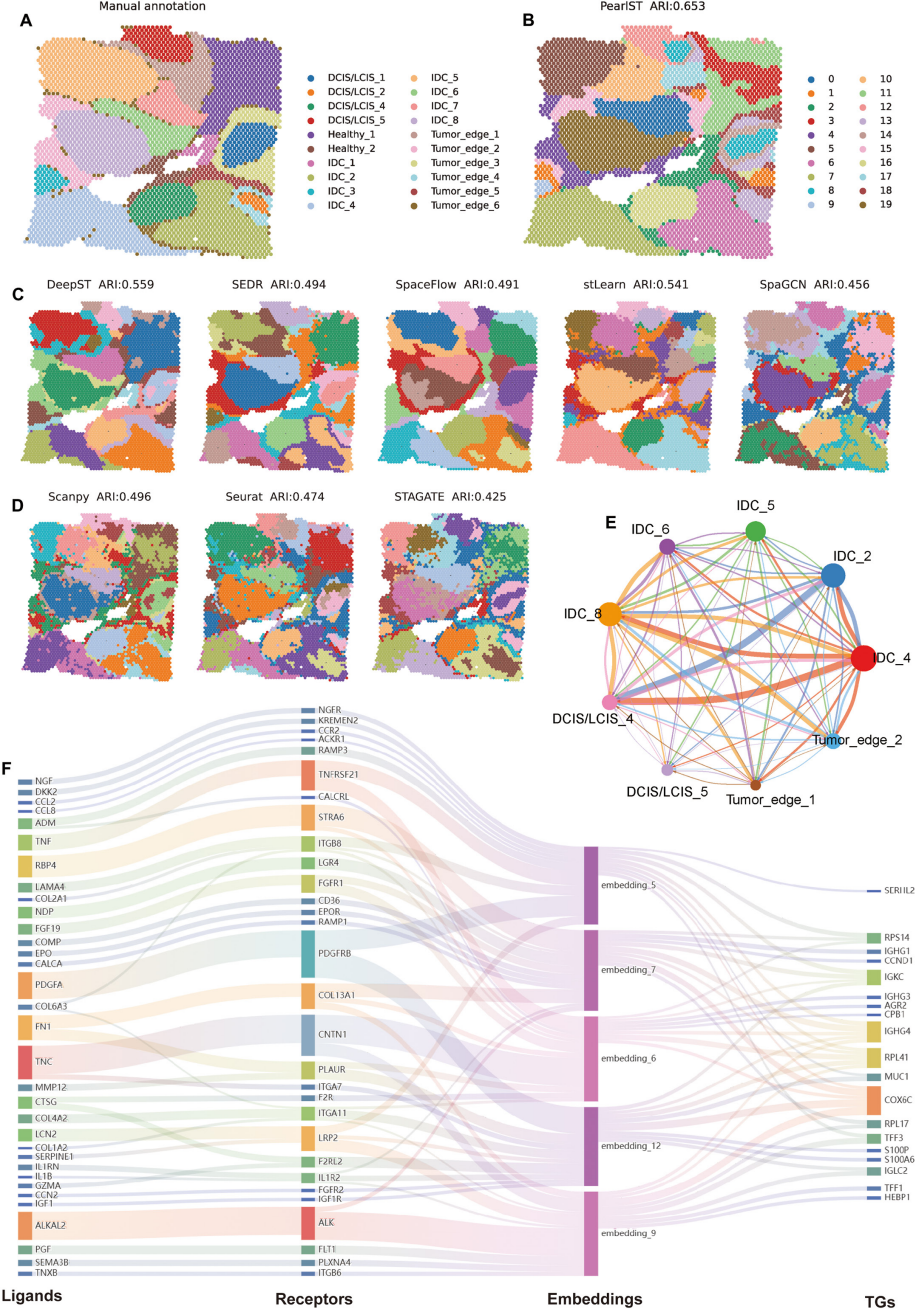
PearlST reveals microenvironment heterogeneity and functional cellular interactions in 10X Visium data of human breast cancer. (A) Manual pathology labeling based on H&E staining from SEDR. (B) Spatial clustering results on human breast cancer by PearlST. (C) Spatial region segmentations and ARI scores generated by DeepST, SEDR, SpaceFlow, stLearn, and SpaGCN. (D) Spatial region segmentations and ARI scores obtained by SCANPY, Seurat, and STAGATE. (E) Visualization of interaction strengths among regions, where the size of the dots reflects the number of spots, and the thickness of the lines indicates the interaction strength. (F) Visualization of the multilayer cellular communication network between region IDC_4 and Tumor_edge_1, where each layer represents ligands, receptors, low-dimensional embeddings, and target genes from left to right.

To investigate the spatial heterogeneity of the cancer microenvironment, we applied PearlST to a 10X Visium ST dataset of human breast cancer. Initially, we identified spatial domains for the human breast cancer dataset and compared the results with manual annotation based on pathological labeling from H&E staining by SEDR [22]. Notably, unlike previous datasets on DLPFC, mouse hippocampus, etc., which have clear and defined morphological boundaries, tumor tissues are highly heterogeneous and contain a complex microenvironment [22,44]. Based on pathological features, the histological image was segmented into 20 regions, consisting of 4 main morphotypes: ductal carcinoma in situ/lobular carcinoma in situ (DCIS/LCIS), healthy tissue (Healthy), invasive ductal carcinoma (IDC), and tumor surrounding regions with low features of malignancy (Tumor edge) (Fig. 6A). We assessed the similarity between clustered results and ground truth using the ARI score, comparing a range of popular spatial clustering methods. For Seurat and SCANPY, many of the computed clusters appeared fragmented and discontinuous (Fig. 6D), while STAGATE, SpaGCN, SpaceFlow, DeepST, and PearlST produced fewer disjointed clusters (Fig. 6B to D). PearlST exhibited the best results in terms of region segmentation accuracy and edge noise, particularly for the identification of Tumor_edge_2, DCIS_LCIS_4, IDC_2, IDC_4, IDC_5, IDC_6, and Healthy_1. These results were also reflected in the ARI scores, with PearlST achieving the highest score at 0.65 (Fig. 6B), while DeepST and stLearn scored under 0.55, and SEDR, SpaceFlow, and SCANPY scored around 0.49. The remaining 3 methods had lower ARI scores (Fig. 6C and D).

Continuing our analysis, we extended PearlST to study cell–cell communication between regions. Initially, we selected 9 regions that were closer to the manual annotations to explore the strength of their interactions (Fig. 6E). On a macro level, stronger interactions were observed between regions DCIS/LCIS_4 and IDC_2, as well as between DCIS/LCIS_4 and IDC_4. Tumor_edge_2 received a significant number of ligand-signaling molecules from IDC_4 and IDC_8. Notably, weaker interaction strengths were observed between regions of the same type, such as IDC, Tumor edge, and DCIS/LCIS (Fig. 6E). Zooming in on a micro level, we studied a multilayer signaling network between any 2 regions. Taking the example of constructing a multilayer communication network between 2 regions IDC_4 and Tumor_edge_1, we employed the law of mass reciprocity to calculate the ligand–receptor (LR) scores between these regions. We then used random forest regression to establish the relationship between LR scores and low-dimensional embeddings of spots (Fig. S12A and B). To further explore the genes contributing significantly to the low-dimensional embeddings, we computed feature importance using stochastic perturbations. Genes identified as significantly contributing genes were considered as target genes in the multilayer network. The role of LR pairs in regulating target genes was reflected by their previous effects on low-dimensional embeddings. The analysis revealed that certain LR pairs, including TNF-TNFRSF21, RBP4-STRA6, PDGFA-PDGFRB, TNC-CNTN1, and ALKAL2-ALK, exhibited stronger interactions (Fig. 6F). In particular, ALKAL2-ALK exerted a dominant regulatory effect on the generation of embedding_9, while PDGFA-PDGFRB regulated embedding_5 and embedding_12 simultaneously (Fig. 6F and Fig. S12C to G).

Lastly, we performed functional enrichment analysis of target genes using clusterProfiler [45], encompassing KEGG (Kyoto Encyclopedia of Genes and Genomes) enrichment (Fig. S13A) and GO (Gene Ontology) enrichment (Fig. S13B). The analysis revealed that the target genes are significantly enriched in numerous signaling pathways associated with cancer development and progression. Notable pathways include the PI3K-AKT signaling pathway [46], p53 signaling pathway [47], immunoglobulin-mediated immune response [48], and B cell receptor (BCR) signaling pathway [49] (Fig. S13). Notably, the abovementioned ALKAL2-ALK signaling [50–53] and PDGFA-PDGFRB signaling [54,55] have been reportedly important in breast cancer growth and metastasis. Moreover, the PI3K-AKT pathway has been validated as the downstream signaling pathways of ALKAL2-ALK signaling and PDGFA-PDGFRB signaling in human cancers [53,56,57]. These findings suggest important functional interactions between the 2 regions IDC_4 and Tumor_edge_1 through LR interactions in the tumor microenvironment. More specifically, from the multilayer and functional regulation network of the learned embedding, one can infer which target genes are important genes contributing to this embedding and how they are regulated by other domains via intercellular and intracellular signaling pathways.

## Discussion

Accurately inferring spatiotemporal relationships of cells or spots from the ST data are pivotal for comprehending tissue structure and biological functionality. In this study, we introduce a diffusion-based adversarial learning approach to identifying spatiotemporal structures from the ST data. The developed tool, PearlST, combines a PDE-based diffusion model and an adversarial graph autoencoder to effectively extract higher-order features and identify spatiotemporal structures from the ST data. Specifically, PearlST integrates spatial location, histology images, and gene expression to define spatial nearest neighbors and constructs a PDE-based diffusion model to enhance expression profiles, and then transforms ST data into low-dimensional embeddings via a Wasserstein adversarial regularized graph autoencoder, capturing both expression similarity and spatial proximity of cells. Based on the learned low-dimensional embeddings, PearlST can perform various downstream analysis, including (a) dissecting spatial domains exhibiting consistent expression patterns, well-defined boundaries, and reduced noise; (b) inferring spatiotemporal states of cells or spots and constructing cell developmental trajectories; and (c) connecting intercellular LR signaling to intracellular target genes established by constructing a multilayer network to interpret biological functions of the latent embeddings. It should be noted that the augmented gene expression profiles are not only used for constructing the graph autoencoder but also used for other downstream analysis tasks such as feature plotting and pseudotime analysis, because the low-dimensional embeddings are generated by a graph autoencoder with augmented gene expression as input.

Such methodological advantages lead to PearlST’s strong capability in analyzing diverse properties of ST datasets generated by different platforms with varying spatial resolutions. Notably, PearlST accurately unveiled the laminar organization of the DLPFC and the MOB. PearlST revealed finer-grained tissue structures of the mouse embryo, clearly identified known tissue structures of the hippocampus, and uncovered spatial domains. When applied to an image-based ST dataset at single-cell resolution generated by STARmap, PearlST demonstrated the highest clustering accuracy and superior results in terms of both region segmentation accuracy and edge noise. The pSM calculated by PearlST exhibited layered patterns consistent with the developmental sequences of the mouse visual cortex tissue, which were not discernible from non-spatial pseudotime analyses. Moreover, PearlST was applied to identify tumor regions in human breast cancer data obtained from 10X Visium, enabling the study of interactions between regions and regulations from LR interactions to target genes. Enrichment analysis of target genes revealed multiple signaling pathways linked to cancer development and progression.

We acknowledge that PearlST has some limitations. Firstly, the current version of PearlST is not very suitable for imaging-based ST data (e.g., MERFISH [12,13]) that are characterized by a small number of genes. At present, we simply modified PearlST as follows. We refrained from employing a diffusion model for denoising. Instead, we solely utilized nearest-neighbor information to augment the transcriptional profile of each cell. Subsequently, K-means was directly applied for spatial domain dissection, while the graph autoencoder was not utilized. More details are described in Note S2. The results demonstrate that the modified PearlST can also be applied to ST data with low coverage. In our forthcoming research, we aim to refine our computational framework, thereby developing a unified version of PearlST capable of accommodating both high-coverage and low-coverage ST data. The second limitation is that for different datasets, different tuning hyperparameters may be needed to achieve the best results. These 3 hyperparameters are “epoch”, “dropout”, and “gp_lambda”. Among them, “epoch” controls the number of network training, “dropout” can prevent model overfitting, and “gp_lambda” is used to balance the loss of each part of the function. In addition, we used different spatial graphs for gene expression augmentation and graph autoencoder in the current version of PearlST, which can be refined by simply using Eq. 3 as a graph input of graph autoencoder.

There are several other aspects for improving our computational framework. First, while compared to other denoising methods, the PDE-based diffusion model has good interpretability and high computational efficiency, one may incorporate deep learning-based augmentation techniques into the framework of PearlST to further enhance the gene expression profiles. Second, based on the pseudotime derived from PearlST, dynamic models can be built to infer gene regulatory networks and cell–cell communication networks along with the temporal trajectories of cell fate development. Third, feature extraction from histological images can be further improved. We have observed that histological morphology plays a crucial role in data enhancement. Many existing methods rely on pre-trained models, such as ResNet50 trained on ImageNet, leading to a significant heterogeneity mismatch between the source and target data. This mismatch limits the reliability of visual features extracted from histological images. In contrast, the approach proposed by Zuo et al. [58], which utilizes contrastive learning to train SimCLR with an encoder network based on ResNet50 for image feature extraction, appears more rational. However, its extended runtime is deemed impractical at this time. For instance, the execution time on any of the DLPFC samples using our NVIDIA GeForce RTX 3060 GPU exceeded 240 min. Here, we adopt a different strategy by training SimCLR with a substantial number of histological images and employing the trained model for target data. This approach not only reduces data heterogeneity but also minimizes the computational time required for feature extraction. Certainly, there may be alternative and potentially more effective methods for processing images, and further effort in this direction is warranted.

In summary, PearlST offers a versatile and effective tool for unraveling intricate spatiotemporal structures in ST data across various biological contexts. It excels in segmenting spatial domains, inferring spatiotemporal patterns of cells within tissues, and constructing functional and multilayer cellular communication networks between cell regions.

## Materials and Methods

### Data preprocessing

The preprocessing of gene expression matrix involves the following steps. Initially, gene filtering is executed to retain only those genes expressed in a minimum of 4 cells or more. Subsequently, normalization occurs, wherein the expression of each gene is divided by the total expression in the respective cell. This normalization ensures that each cell attains an identical total count after the process. The normalized expression is then multiplied by a scale factor (default 10,000) and subjected to log-transformation with a pseudo-count of one. Lastly, the top 2,000 highly variable genes are selected as the inputs for PearlST. The above procedures were implemented using the SCANPY package.

### Integrating gene expression, histology image, and spatial locations to compute spatial nearest neighbors

Spatial gene expression technology offers transcriptome-wide gene expression profiles, accompanied by spatial position information and/or tissue morphology. In the case of PearlST, these additional tissue data are leveraged to enhance gene expression in adjacent spots. PearlST employs ResNet-50 [29] trained through the SimCLR framework [30] to learn visual features from each spot image. The training dataset comprises histology images collected from various tissues of different species. PearlST evaluates gene expression similarity, morphological similarity, and spatial relationships between each pair of spots. The gene expression weight between spot *i* and spot *j* (*ES_ij_*) was computed using the cosine similarity as follows:

$${\mathrm{ES}}_{\mathrm{ij}}=\frac{\left(G_{i}-{\bar{G}}_{i}\right)\cdot \left(G_{j}-{\bar{G}}_{j}\right)}{{\left\|G_{i}-{\bar{G}}_{i}\right\|}_{2}\cdot {\left\|G_{j}-{\bar{G}}_{j}\right\|}_{2}}$$ (1)

where *G_i_* and ${\bar{G}}_{i}$ denote the expression profiler and the mean expression of spot *i*, respectively. Following this, the morphological similarity between spot *i* and the adjacent spot *j* (*MS_ij_*) was computed as follows:

$${\mathrm{MS}}_{\mathrm{ij}}=\frac{M_{i}\cdot M_{j}}{{\left\|M_{i}\right\|}_{2}\cdot {\left\|M_{j}\right\|}_{2}}$$ (2)

where *M_i_* refers to the visual features of spot image *i*, with each spot image transformed into 2,048 dimensional latent variables. Subsequently, the spatial distance between the considered cell and all other cells is calculated. The distances between the top 30 (optional) adjacent spots are then ordered to determine the radius *γ* (mean add variance). For a given spot *i*, a spot *S_j_* is considered to be a neighbor, then *PS_ij_* = 1 if and only if the Euclid distance between the 2 spots is less than *γ*; otherwise, *PS_ij_* = 0. Finally, under the combined consideration of spatial gene expression, histological similarity, and spatial adjacency, the overall pseudo similarity (OS) between spots *i* and *j* can be expressed as:

$$OS_{\mathrm{ij}}={\mathrm{ES}}_{\mathrm{ij}}\cdot {\mathrm{MS}}_{\mathrm{ij}}\cdot {\mathrm{PS}}_{\mathrm{ij}}$$ (3)

We denote by *NN_i_* the set consisting of the top (default by 4) nearest neighbors of spot *i* according to Eq. 3. In this way, we integrate multiple pieces of information to more precisely calculate the spatial nearest neighbors of each cell or spot.

### Gene expression augmentation using the PDE-based diffusion model

Given the inherent noise in spatial transcriptome data, we utilized a PDE-based diffusion model to both denoise and enhance gene expression. This enhancement is achieved by considering the spatial nearest neighbors of each spot, as obtained in the previous section. Here, the transcriptional profile of log-transformed gene expression for each spot is represented by *u*(*x*, *y*) = *u*(*x*, *y*, *t*)|_*t*=0_ in a bounded region Ω. Then, the following PDE model is employed to describe the diffusion process of *u*(*x*, *y*),

∂u∂t=∇·c∇u∇u,x,y,t∈Ω×0,∞ux,y,0=ux,y,x,y∈Ω∂u∂n→=0,x,y,t∈∂Ω×0,∞ (4)

where *c*(|∇*u*|) is referred to as the diffusion function, which is a function of the gradient mode of the image. In other words, the smoothing operator of this diffusion model varies with the changes in the magnitude of the gradient. For this purpose, we adopt a diffusion function proposed by Perona and Malik [59]:

$$c\left(\left|\nabla \;u\right|\right)=\frac{1}{1+{\left(\frac{\left|\nabla u\right|}{K}\right)}^{2}}$$ (5)

where *K* is a constant, taken by default to be 0.2. Discretizing Eq. 4 gives its iterative difference form as:

$$u_{i,j}^{n+1}=u_{i,j}^{n}+\lambda \left[c_{i+1,j}^{n}\nabla Su_{i,j}^{n}+c_{i,j+1}^{n}\nabla Eu_{i,j}^{n}+c_{i-1,j}^{n}\nabla Nu_{i,j}^{n}+c_{i,j-1}^{n}\nabla Wu_{i,j}^{n}\right]$$ (6)

In the above equation, $\nabla \;Su_{i,j}^{n}\;=\;u_{i+1,j}^{n}\;-u_{i,j}^{n}$, $\nabla \;Eu_{i,j}^{n}\;=\;u_{i,j+1}^{n}\;-u_{i,j}^{n}$, $\nabla \;Nu_{i-1,j}^{n}=u_{i-1,j}^{n}-u_{i,j}^{n}$, $\nabla \;Wu_{i,j}^{n}=u_{i,j-1}^{n}-u_{i,j}^{n}$, and *λ* is a small positive parameter, which, in this article, defaults to 0.1. See Note S3 for a more detailed derivation. Prior to data augmentation, the transcriptional profile of each spot is reshaped to a 50 × 40 gray scale plot. Gene expression denoising is achieved by iteratively updating Eq. 6, where $u_{i,j}^{n}$ denotes the pixel value at grid (*i, j*) and *n* represents iteration times. Next, gene expression augmentation is performed by substituting the gene expression of the corresponding spatial nearest neighbors of each spot obtained by Eq. 3 into Eq. 6 for iteration. This means that $\nabla \;Su_{i,j}^{n}$, $\nabla \;Eu_{i,j}^{n}$, $\nabla \;Nu_{i-1,j}^{n}$, and $\nabla \;Wu_{i,j}^{n}$ are computed using the expressions of the nearest neighbors belonging to *NN_i_*. The number of iterations is set to 4 in this study, which reconciles both prediction performance and computational efficiency.

### Wasserstein adversarial regularized graph autoencoder

#### Spatial expression graph construction

We construct a spatial expression graph (SEG) utilizing the augmented gene matrix of highly variable genes. The SEG is formed based on the spatial proximity of cells, where nodes represent cells or spots, and edges characterize the spatial relationships between these cells or spots. The SEG comprises 2 matrices, count matrix *X* = {*x*_1_, *x*_2_, ⋯*x_N_*} and spatial adjacency matrix *A* ∈ *R*^*N*×*N*^, where *N* is equal to the number of cells or spots and *A_ij_* = 1 if node *i* and *j* are connected by an edge; otherwise, *A_ij_* = 0.

In this process, we adopt an alpha complexity-based approach to build a SEG. Initially, a Voronoi cell is generated for each cell or spot located at *r* as:

$$V\left(r\right)=\left\{x\in R^{2};\left\|x-r\right\|\leq \left\|x-r^{\prime }\right\|,\forall r^{\prime }\in C\right\},$$ (7)

where *C* represents the set of coordinates for all the cells or spots, and ‖·‖ denotes the Euclidean distance. Subsequently, the 1-skeleton of the alpha complex [60] is employed to identify the neighborhood edges E of the spots, expressed as follows:

$$E=\left\{\left(i,j\right)|\cap_{k\in \left(i,j\right)}\left(V\left(r_{k}\right)\cap Β\left(r_{k},\delta \right)\right)\right\},$$ (8)

where Β(*x*, *δ*) is a circular region in *R*^2^ centered at *x* with radius *δ*, and *k* ∈ (*i*, *j*) represents the *k*th nearest neighbor at spatial position (*i*, *j*). *k* was set to 30 in our study. The radius *δ* is estimated from the average distance of the *k*-nearest neighbors of the spot.

#### Graph autoencoder

Given the SEG *G*, we utilize a 2-layer graph convolutional network as generator Φ*_ω_*(*A*, *X*) to encode the original node features *X* ∈ *R*^*N*×*d*^ with the topological structure *A* into a low-dimensional representation *Z* ∈ *R*^*N*×*d*^′^^:

$$\Phi_{\omega }\left(A,X\right)=\text{ReLU}\left(\bar{A}\cdot \text{ReLU}\left(\bar{A}XW_{1}\right)\cdot W_{2}\right),$$ (9)

where $\bar{A}={\tilde{D}}^{-\frac{1}{2}}\tilde{A}{\tilde{D}}^{-\frac{1}{2}}$ is the new “weighted” adjacency matrix for graph *G* after convolution, and the default value for the number of low dimensions *d*′ is 32. ${\tilde{D}}_{\mathrm{ii}}=\sum_{j}{\tilde{A}}_{\mathrm{ij}}$ is the degree matrix with $\tilde{A}=A+I$, *I* is a unit matrix. *ReLU*(*h*) =  max (0, *h*) is used as the activation function in the neural networks and the output matrix *Z* contains latent embeddings *z_i_* for each node *v_i_* ∈ *V* as row vectors. We assume that the low-dimensional representation follows a standard Gaussian distribution *N*(0, *I*) denoted by *P_r_* and define the Φ*_ω_* generated low-dimensional embedding as *P_g_*(*Z*| *A*, *X*), followed by reconstructing the adjacency matrix to learn the parameter *ω* using an inner product decoder:

$$\begin{array}{c} p\left(A|,\;X,Z\right)=\prod_{i=1}^{n}\prod_{j=1}^{n}p\left(A_{\mathrm{ij}}|z_{i},z_{j}\right),\\ p\left(A_{\mathrm{ij}}=1|z_{i},z_{j}\right)=\sigma \left(z_{i}^{T}z_{j}\right). \end{array}$$ (10)

Here, the sigmoid activation function is used to make the decoded data in the range from 0 to 1 and the generator is optimized using the cross-entropy loss function:

$$\underset{\omega }{\text{argmin}}-E_{P_{g}}\left[\text{log}p\left(A|X,Z\right)\right],$$ (11)

where *E_P_g__* refers to the expectation of the distribution *P_g_*.

#### Wasserstein regularizer

To force the encoded distribution *P_g_*(*Z*| *A*, *X*) into the target distribution *N*(0, *I*), we introduce a Wasserstein regularizer that helps to minimize the 1-Wasserstein distance between *P_r_* and *P_g_* [31]

$$\begin{array}{c} W_{1}\left(\;P_{r},P_{g}\;\right)=\underset{\phi \in \Phi }{\text{max}}\left\{E_{r\sim P_{r}}\left[f_{\phi }\left(r\right)\right]-E_{z\sim P_{g}}\left[f_{\phi }\left(z\right)\right]\right\} \end{array}$$ (12)

*f_ϕ_* in the above equation is a multilayer perceptron (MLP) containing 2 hidden layers:

$$f_{\phi \in \Phi }\left(z\right)=W_{5}\sigma \left(W_{4}\sigma \left(W_{3}z+b_{1}\right)+b_{2}\right)+b_{3}$$ (13)

where *W*_3_, *b*_1_ and *W*_4_, *b*_2_ are weights and biases of the 2 hidden layers, and *W*_5_, *b*_3_ correspond to the output layer of MLP. This also leads to an adversarial learning framework with *minimax* objective:

$$\underset{\omega }{\text{min}}\;\underset{\phi \in \Phi }{\text{max}}\left\{E_{r\sim P_{r}}\left[f_{\phi }\left(r\right)\right]-E_{z\sim P_{g}}\left[f_{\phi }\left(z\right)\right]\right\}$$ (14)

Combined with the reconstruction loss during the training generator phase, the final loss function of the graph autoencoder is defined as:

$$\begin{array}{c} \underset{\omega }{\text{min}}\;\underset{\phi \in \Phi }{\text{max}}\left\{-E_{P_{g}}\left[\text{logp}\left(A|X,Z\right)\right]+E_{r\sim P_{r}}\left[f_{\phi }\left(r\right)\right]-E_{z\sim P_{g}}\left[f_{\phi }\left(z\right)\right]\right\} \end{array}$$ (15)

Finally, the low-dimensional embeddings outputted by the graph autoencoders were applied to domain segmentation, pSM construction, spatial trajectory inference, and low-dimensional visualizations.

### Domain segmentation and pSM computing

The domain segmentations are acquired by applying K-means to the low-dimensional embeddings from PearlST. The pSM is computed by employing the DPT algorithm [33], utilizing the low-dimensional representations output from PearlST. DPT employs diffusion-like random walks to estimate the ordering and transitions between cells. By utilizing the embeddings from PearlST, which encode both spatial location and gene expression profiles of cells, DPT generates a spatiotemporal order that is consistent in both space and pseudotime. The root cell of the pSM can be specified with a priori knowledge; otherwise, in our strategy, the cell in the embedding space that has the largest sum distance from the other cells is assigned as the root cell.

#### Spatial trajectory inference

We utilize the PAGA algorithm [61], implemented in the SCANPY package, to illustrate spatial trajectories. The low-dimensional representation outputted by PearlST serves as input for PAGA, which constructs a topological map of relationships between cells by analyzing transcriptome similarities. The PAGA algorithm then leverages this topological map to infer the developmental trajectory of cells and calculate the expression dynamics of each gene along the trajectory.

### Inference of functional and multilayer cellular communication networks

Building on our prior work stMLnet [62], we quantify spatially dependent LR signaling activity and infer downstream functions by associating LR pairs to the latent features learned by PearlST and target genes. Specifically, the spatial distance-dependent LR signaling scores can be quantified based on a diffusion model and the law of mass action. After obtaining LR scores, we employed Random Forest regression to learn the regulatory relationship between LR pairs and the low-dimensional representation of receiver cells. Additionally, we computed the feature importance of the graph autoencoder by random perturbations identifying those genes that contribute more to the low-dimensional representations and considering them as target genes. This process allowed us to construct a multilayered signaling network (ligand–receptor–embeddings–target genes) for dissecting cellular communications between different spatial domains. Furthermore, enrichment analysis (e.g., KEGG or GO enrichment) of target genes can be performed using tools like clusterProfiler [45] to elucidate the biological functions of the LR-mediated cellular interactions. For a more detailed description of the inference of functional and multilayer cellular communication networks, please refer to Note S4.

## Data Availability

All datasets analyzed in this paper are publicly available. Specifically, the DLPFC dataset is accessible within the spatialLIBD package (http://spatial.libd.org/spatialLIBD). The processed Stereo-seq data from MOB tissue6 is accessible at https://github.com/JinmiaoChenLab/SEDR_analyses, and the other Stereo-seq data from mouse embryo data at E12.5 can be downloaded from https://db.cngb.org/stomics/mosta/ or the SODB database (https://gene.ai.tencent.com/SpatialOmics/). The Slide-seqV2 dataset from MOB tissues is available at the Broad Institute Single Cell Portal at https://singlecell.broadinstitute.org/single_cell/study/SCP815/highly-sensitive-spatial-transcriptomics-at-near-cellular-resolution-with-slide-seqv2#study-summary, and mouse hippocampus tissue acquired by Slide-seqV2 can be downloaded from https://portals.broadinstitute.org/single_cell/study/slide-seq-study. Human breast cancer is obtained from the publicly available 10x Genomics Data Repository (https://www.10xgenomics.com/resources/datasets/human-breast-cancer-block-a-section-1-1-standard-1-1-0). The 4i datasets are from https://github.com/scverse/squidpy. The mouse visual cortex STARmap data and MERFISH molecular data are accessible on https://gene.ai.tencent.com/SpatialOmics/. A Python package of PearlST is available from https://github.com/SunXQlab/PearlST. The source codes used for analysis in this study are uploaded on https://github.com/SunXQlab/PearlST-Code.

## Supplementary Materials

Supplementary 1 Notes S1 to S4 Figs. S1 to S13
