# Supplementary material for: Dissecting Spatiotemporal Structures in Spatial Transcriptomics via Diffusion-Based Adversarial Learning
Source: Research (Wash D C). 2024 May 29;7:0390. doi: 10.34133/research.0390 (PMC11134684; doi:10.34133/research.0390)
Supplement: Supplementary 1 — Notes S1 to S4 Figs. S1 to S13 [file research.0390.f1.pdf]

Supplementary Information

Dissecting Spatiotemporal Structures in Spatial Transcriptomics via Diffusion-based Adversarial Learning

Haiyun Wang[1,†], Jianping Zhao[1,†], Qing Nie[2], Chunhou Zheng[3,*], Xiaoqiang Sun[4,*]

[1]College of Mathematics and System Sciences, Xinjiang University, Urumqi, China.
[2]Department of Mathematics and Department of Developmental & Cell Biology, NSF-Simons Center for Multiscale Cell Fate Research, University of California Irvine, Irvine, USA.
[3]School of Artificial Intelligence, Anhui University, Hefei, China.
[4]School of Mathematics, Sun Yat-sen University, Guangzhou, China.

***Correspondence to:** Chunhou Zheng (zhengch99@126.com) and Xiaoqiang Sun (sunxq6@mail.sysu.edu.cn)

This file includes the following subsections:

• Supplementary Notes

• Supplementary Figures

Supplementary Note S1

Implementation of comparison methods

We ran each method according to the official technical documentation and used the default parameters for each method without making any modifications to the models. Here, we provide the link of the tutorial for each comparison method.

- The STAGATE's tutorials are at https://stagate.readthedocs.io/en/latest/.
- The BayesSpace can be obtained via https://github.com/edward130603/BayesSpace.
- The DeepST is at https://github.com/JiangBioLab/DeepST.
- The tutorials of SpaGCN are at https://github.com/jianhuupenn/SpaGCN/blob/master/tutorial/tutorial.ipynb.
- The tutorials of stLearn are at https://stlearn.readthedocs.io/en/latest/.
- The usage instructions for SEDR are at https://sedr.readthedocs.io/en/latest/.
- The SpaceFlow's results can be generated according to https://github.com/hongleir/SpaceFlow.
- The SpecMix can be accessed via https://github.com/ma-compbio/SpiceMIx.
- The Scanpy is at https://github.com/scverse/scanpy
- The Seurat is at https://github.com/satijalab/seurat

Supplementary Note S2

Modified PearlST tailored for MERFISH and 4i datasets

Beyond the 10X Genomics Visium platform, we explored the generalization capability of PearlST on imaging-based molecular data, specifically MERFISH [3, 4], and 4i (iterative indirect immunofluorescence imaging) [5]. In this context, the PDE-based diffusion model integrated into PearlST was utilized for ST data augmentation. Initially, we applied PearlST to MERFISH data obtained from the SODB database [6], which houses various ST datasets generated by multiple sequencing technologies. Due to the limited number of genes in the MERFISH data (e.g., Moffitt2018Molecular [3] contains 161 genes expressed on 5693 cells, and Allen2022Molecular [7] contains 374 genes expressed on 9665 cells), we refrained from employing a diffusion model for denoising. Instead, we solely utilized nearest neighbor information to augment the transcriptional profile of each cell. Subsequently, K-means was directly applied for spatial domain dissection, while the graph autoencoder was not utilized. For Allen2022Molecular, which contains 13 cell types, the ARI score of K-means between clustering results and the ground truth on the raw data was 0.72, increasing to 0.80 after data augmentation by the diffusion model (fig. S9A). Similarly, Moffitt2018Molecular, which contained 16 cell types, showed an increase in ARI score from 0.37 to 0.42 following data augmentation (fig. S9B). Subsequent to the MERFISH evaluation, we assessed the performance of PearlST in data augmentation on 4i data, which measured 40 protein reads in high-throughput biological samples ranging from millimeter to nanometer scales (~270,000 observations/pixels). Here, only partial molecular data were used for spatial domain identification. Gene expression augmentation improved the clustering performance of 4i_119 [5] from 0.55 to 0.59 and 4i_122 [5] from 0.43 to 0.47 (fig. S9C-D). These results demonstrate that the modified PearlST can also be applied to ST data with low coverage. In our forthcoming research, we aim to refine our computational framework, thereby developing a unified version of PearlST capable of accommodating both high-coverage and low-coverage ST data.

Supplementary Note S3

PDE-based diffusion model for SRT data augmentation

We employed a partial differential equation (PDE)-based diffusion model [1] to denoise and enhance gene expression using spatial nearest neighbors of each spot obtained in the previous section. Here, the transcriptional profile of log-transformed gene expression of each spot and its spatial nearest neighbors is viewed as an image. Let the image be defined in a bounded region Ω and the initial noise image be $u(x,y) = u(x,y,t)|_{t=0}$. The diffusion process can be represented by the following PDE model:

$$\begin{cases} \frac{\partial u}{\partial t} = \nabla \cdot (c(|\nabla u|)\nabla u), & (x,y,t) \in \Omega \times (0,\infty) \\ u(x,y,0) = u(x,y), & (x,y) \in \Omega \\ \frac{\partial u}{\partial \vec{n}} = 0, (x,y,t) & (x,y,t) \in \Omega \times (0,\infty) \end{cases} . \quad (1)$$

$c(|\nabla_u|)$ is called the diffusion function, which is a function of the gradient mode of the image, in other words, the smoothing operator of this diffusion model varies with the changes in the magnitude of the gradient. The choice of the diffusion function is most critical for the diffusion extremes, Perona and Malik [1] proposed that the diffusion function can take the following two forms:

$$c_1(|\nabla u|) = \frac{1}{1+\left(\frac{|\nabla u|}{K}\right)^2}, \text{ or } c_2(|\nabla u|) = exp\left[-\left(\frac{|\nabla u|}{K}\right)^2\right]. \quad (2)$$

Eq. (1) can be expanded as follows,

$$\frac{\partial u}{\partial t} = \nabla \cdot (c(|\nabla u|)\nabla u) = c(|\nabla u|)\Delta u + \nabla c(|\nabla u|) \cdot \nabla u. \quad (3)$$

When $c(|\nabla u|) = c_1(|\nabla u|)$,

$$\frac{\partial u}{\partial t} = \frac{1-\frac{|\nabla u|^2}{K^2}}{1+\frac{|\nabla u|^2}{K^2}} \cdot u_{\xi\xi} + \frac{1}{1+\frac{|\nabla u|^2}{K^2}} \cdot u_{\eta\eta}. \quad (4)$$

The constant in the diffusion function K value plays an important role in the denoising of the image. Therefore, the diffusion characteristics of the P-M model can be summarised as follows: the diffusion coefficients in the tangential and normal directions in the regions where $|\nabla_u| \leq K$ are the same, which is isotropic diffusion; while the diffusion coefficients in the regions where $|\nabla_u| \geq K$ are zero, i.e., there is no smoothing, and thus the edges of the image are preserved.

In the following we present the discrete algorithm for the PM model. We firstly use the finite difference method to compute the difference equation, and then use the iterative method to obtain the approximate solution of the difference equation. The iterative format is also very concise, and the small computational cost is one of the important advantages of the P-M model. The derivation process of the algorithm is analysed below.

Let the size of the image u be $(N+1) \times (N+1)$, then $u(i,j)$ is the grey value obtained at coordinate (i,j). Dividing the time factor t into n parts and setting the time interval Δt to be small enough, the time factor is $t = n \cdot \Delta t$. A Taylor series expansion of X gives:

$$u(x,y,\Delta t) \approx u(x,y,0) + \Delta t\left(\frac{\partial u}{\partial t}\right)_{t=0} \tag{5}$$

$$= u(x,y,0) + \Delta t \nabla \cdot [c(x,y,0)\nabla u]. \tag{6}$$

Thus, t_{n+1} can be obtained at t_n:

$$\begin{aligned} u(x,y,t_{n+1}) &\approx u(x,y,t_n) + \Delta t \nabla \cdot [c(x,y,t_n)\nabla u(\mathrm{x},\mathrm{y},t_n)] \\ &= u(x,y,t_n) + \Delta t[\nabla(c(x,y,t_n)\nabla u(\mathrm{x},\mathrm{y},t_n) + \\ &\quad c(x,y,t_n)\Delta u(\mathrm{x},\mathrm{y},t_n)] \\ &= u(x,y,t_n) + \Delta t(I_1^n + I_2^n). \end{aligned} \tag{7}$$

Rewrite $u(x,y,t_n)$ in the above equation in discrete format $u_{i,j}^n$, rewrite $c(x,y,t_n)$ to $c_{i,j}^n$. Therefore, the differential form of I_1^n is:

$$\begin{aligned} I_1^n = \frac{1}{2}[&(c_{i+1,j}^n - c_{i,j}^n)(u_{i+1,j}^n - u_{i,j}^n) + (c_{i,j+1}^n - c_{i,j}^n)(u_{i,j+1}^n - u_{i,j}^n) \\ &+(c_{i,j}^n - c_{i-1,j}^n)(u_{i,j}^n - u_{i-1,j}^n) + (c_{i,j}^n - c_{i,j-1}^n)(u_{i,j}^n - u_{i,j-1}^n)] \end{aligned} \tag{8}$$

Similarly the differential form of I_2^n is:

$$I_2^n = \frac{1}{2}c_{i,j}^n\left[u_{i+1,j}^n + u_{i-1,j}^n + u_{i,j+1}^n + u_{i,j-1}^n - 4u_{i,j}^n\right], \tag{9}$$

The two equations are added together to get:

$$I_1^n + I_2^n = \frac{1}{2}\left[c_{i+1,j}^n \nabla S u_{i,j}^n + c_{i,j+1}^n \nabla E u_{i,j}^n + c_{i-1,j}^n \nabla N u_{i,j}^n + c_{i,j-1}^n \nabla W u_{i,j}^n\right], \tag{10}$$

where

$$\nabla S u_{i,j}^n = u_{i+1,j}^n - u_{i,j}^n, \tag{11}$$

$$\nabla E u_{i,j}^n = u_{i,j+1}^n - u_{i,j}^n, \tag{12}$$

$$\nabla N u_{i-1,j}^n = u_{i-1,j}^n - u_{i,j}^n, \tag{13}$$

$$\nabla W u_{i,j}^n = u_{i,j-1}^n - u_{i,j}^n. \tag{14}$$

The final iterative difference form of the Perona-Malik algorithm is obtained as:

$$u_{i,j}^{n+1} = u_{i,j}^n + \lambda\left[c_{i+1,j}^n \nabla S u_{i,j}^n + c_{i,j+1}^n \nabla E u_{i,j}^n + c_{i-1,j}^n \nabla N u_{i,j}^n + c_{i,j-1}^n \nabla W u_{i,j}^n\right], \tag{15}$$

where λ is a small parameter greater than zero, which in this article defaults to 0.1.

Supplementary Note S4

Inference of functional and multilayer cellular communication networks

Based on our previous work [2], we quantify spatially-dependent Ligand-Receptor (LR) signaling activity and infer its downstream functions by linking LR pairs to the latent features learned by PearlST and target genes. More specifically, based on a diffusion model and the law of mass-action, the signaling strength of the k-th LR pair activated at the j-th cell is defined as:

$$LR_j^k = \sum_{i=1}^{n} \left(\frac{1}{d_{ij}} L_i^k R_j^k \right), \tag{16}$$

where d_{ij} represents the distance between cell *i* and cell j. Given LR scores, we then employed Random Forest (RF) regression to learn the regulatory relationship between LR pairs and the low-dimensional representation of receiver cells. Assuming that the low-dimensional embeddings have d' dimensions and that there are n_t ligand-receptor (LR) pairs connected to a given embedding, we decompose the regulatory relationship between the LR pairs and the low-dimensional embeddings into a number of subproblems:

$$Emb_t = f_t\left(LR_1, LR_2, \cdots, LR_{n_t}\right),\ t = 1,2, \cdots d'. \tag{17}$$

Here, we used a tree-based regression model to learn f_t. We used the signaling activities $LR_1, LR_2, \cdots LR_{n_t}$ (calculated from Eq.16) across the receiver cells as input to predict the expression of the embedding Emb_t. d' refers to the dimension of low-dimensional space. Further, we calculated feature importance and rank LR pairs.

Next, we used random perturbations to find those genes that contribute more to the low-dimensional representation, which are used as target genes. For example, for a particular gene, we simulate a knockout by setting its expression profile as a 0 vector. The difference between the low-dimensional representations before and after the perturbation can be expressed as:

$$I_{g_m} = \frac{1}{d'} \left| Emb_n^r - Emb_n^p \right|. \tag{18}$$

The g_m represents the expression of *m-th* gene, Emb_n^r indicates the *n-th* embedding before perturbation, and Emb_n^p is under perturbation. The larger the value of I_{g_m}, the more we consider the contribution of g_m to the *n-th* embedding. In this way, for each embedding of the low-dimensional embeddings, we can find those genes that contribute more to it.

Finally, we used the Wilcoxon test to select the top 5 low-dimensional embeddings with significant differences. Combining the previously mentioned genes that contributed more to these 5 low-dimensional embeddings and the ligand-receptor pairs with significant regulatory relationships, we established a functional and multilayer cellular communication network, i.e., ligand-receptor-embeddings-target genes.

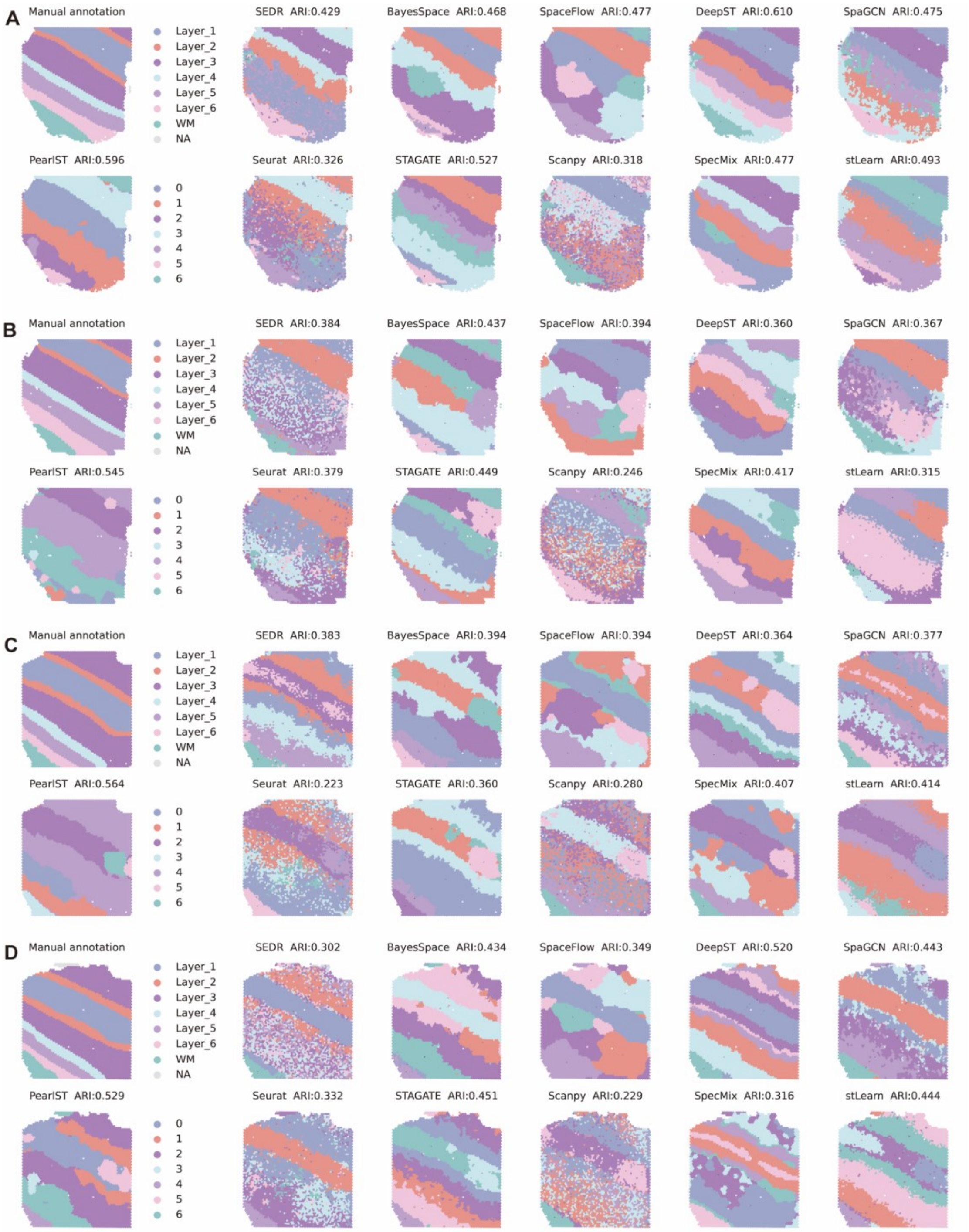

Supplementary Fig. S1. Benchmarking on sections 151507, 151508, 151509 and 15110 using LIBD human dorsolateral prefrontal cortex (DLPFC) SRT data. (A) The results of each method's domain segmentation on the 151507 section are visualized and compared with the Manual annotation results. (**B)** Benchmarking on sections 151508. **(C)** Benchmarking on sections 151509. **(D)** Benchmarking on sections 151510.

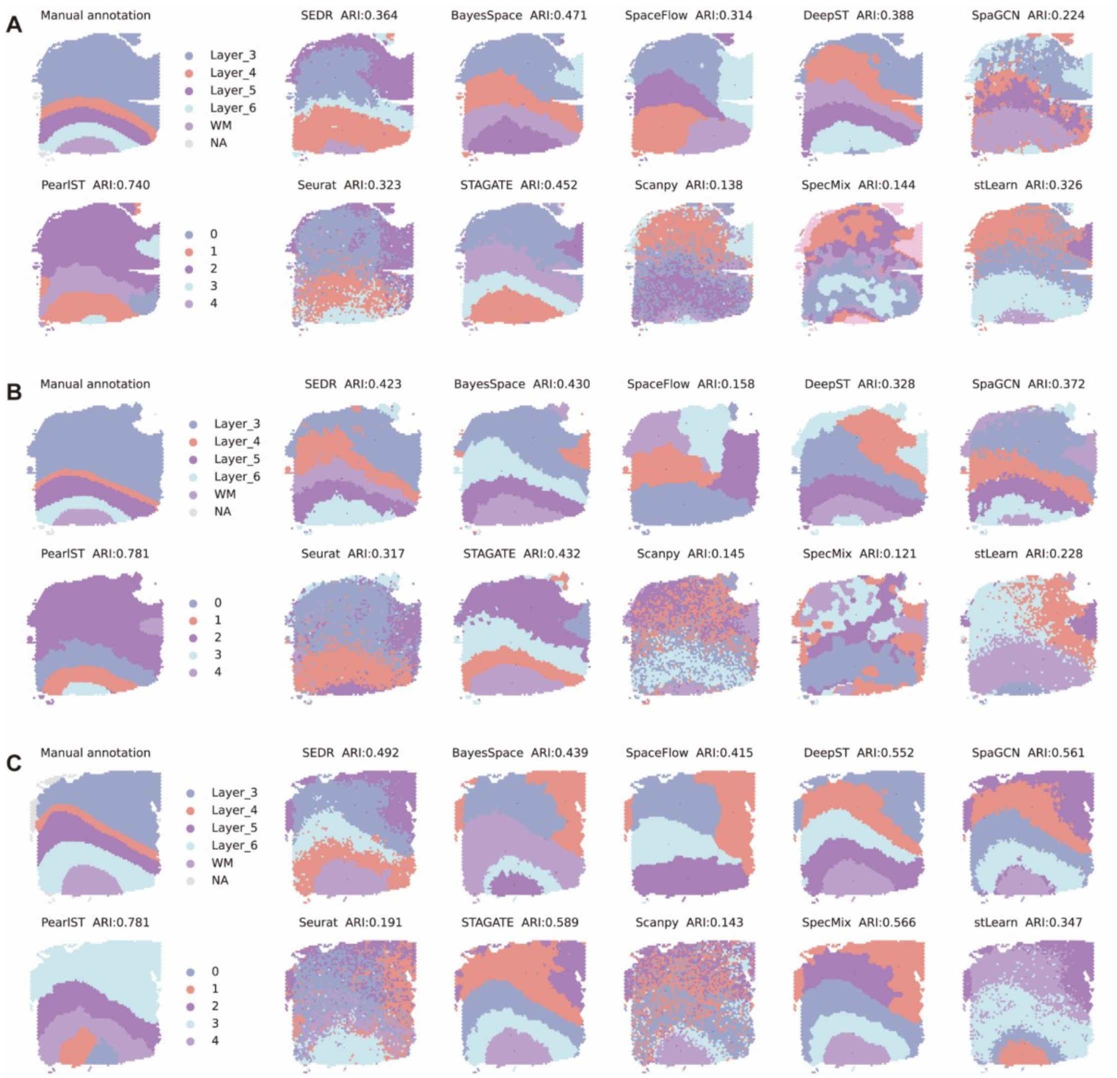

Supplementary Fig. S2. Benchmarking on sections 151669, 151670 and 151672 using LIBD human dorsolateral prefrontal cortex (DLPFC) SRT data. (A) Manual annotations and comparison of spatial domains identified by Seurat, Scanpy, SpaGCN, SpaceFlow, DeepST, BayesSpace, STAGATE, SEDR, SpecMix, stLearn and PearlST on section 151669 of DLPFC dataset. **(B)** Comparison results of 11 methods on section 151670. **(C)** Comparison results of 11 methods on section 151672.

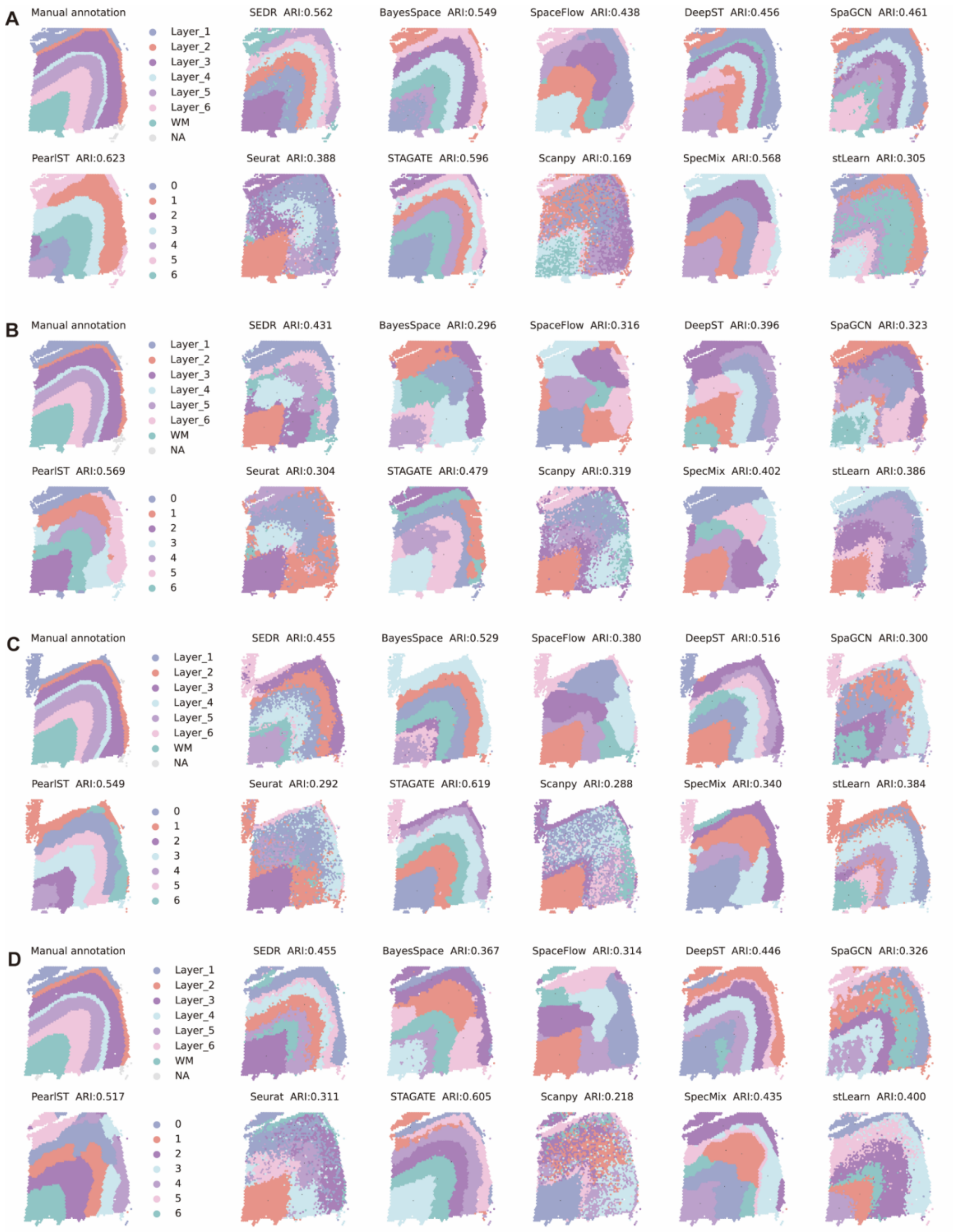

Supplementary Fig. S3. Benchmarking on sections 151673, 151674, 151675 and 151676 using LIBD human dorsolateral prefrontal cortex (DLPFC) SRT data. (A) Manual annotations and comparison of spatial domains identified by Seurat, Scanpy, SpaGCN, SpaceFlow, DeepST, BayesSpace, STAGATE, SEDR, SpecMix, stLearn and PearlST on section 151673 of DLPFC dataset. **(B)** Comparison results of 11 methods on section 151674. **(C)** Comparison results of 11 methods on section 151675. **(D)** Comparison results of 11 methods on section 151676.

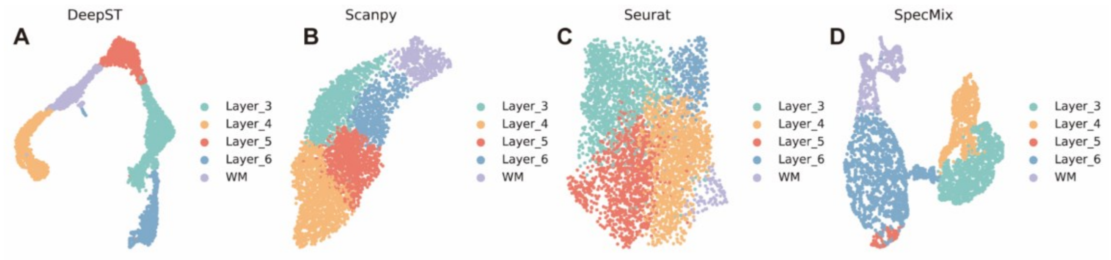

Supplementary Fig. S4. UMAP visualizations generated by low-dimensional representations. UMAP plots corresponding to the latent low-dimensional embeddings outputted by DeepST (A), Scanpy (B), Seurat (C) and SpecMix (D).

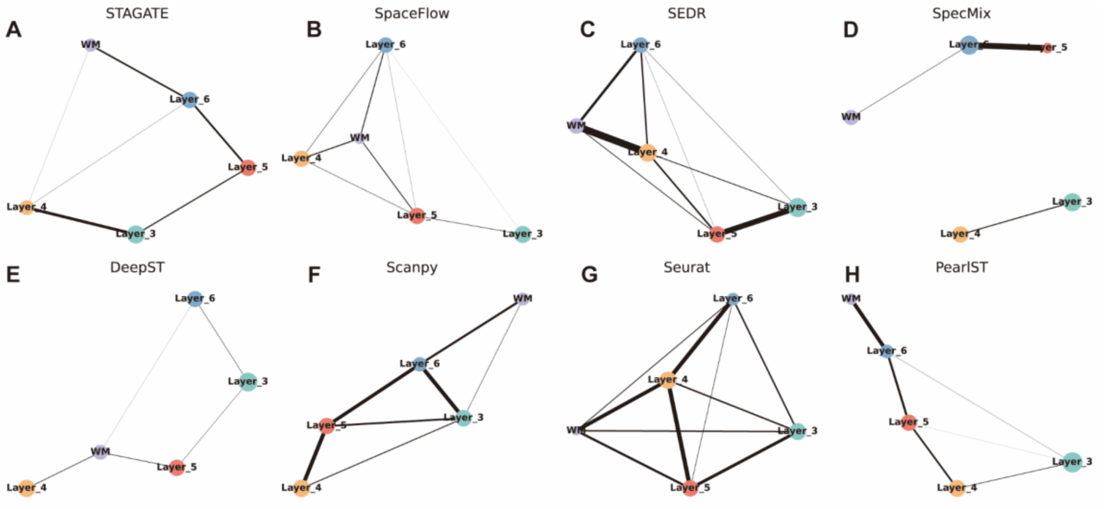

Supplementary Fig. S5. Visualizations of PAGA trajectory inference calculated by low-dimensional representations. PAGA plots corresponding to the latent low-dimensional embeddings outputted by STAGATE (A), SpaceFlow (B), SEDR (C), SpecMix (D), DeepST (E), Scanpy (F), Seurat (G) and PearlST (H). The developmental order of all plots is from left to right, with different coloured dots corresponding to different spatial domains, and the thickness of the lines indicating the strength of relationship or similarity between cells. Thicker lines indicate stronger connections between cells, indicating that they are more related or similar during development or evolution.

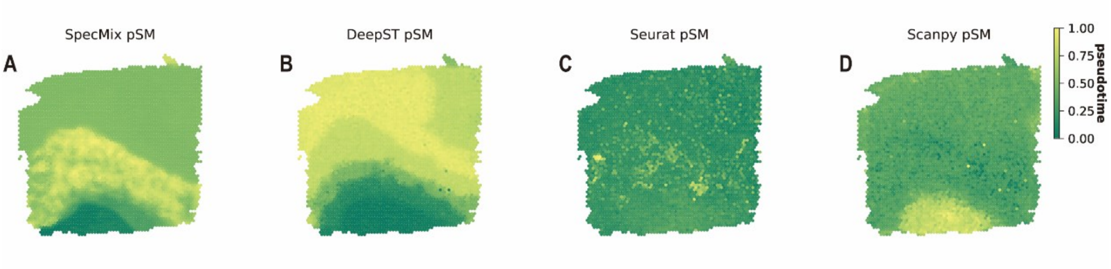

Supplementary Fig. S6. Visualizations of pseudo-spatiotemporal maps. (A) The pSM calculated with the low-dimensional representations outputted by SpecMix. **(B)** The pSM result of DeepST. **(C)** The pSM corresponding to Seurat. **(D)** Scanpy's pSM visualization.

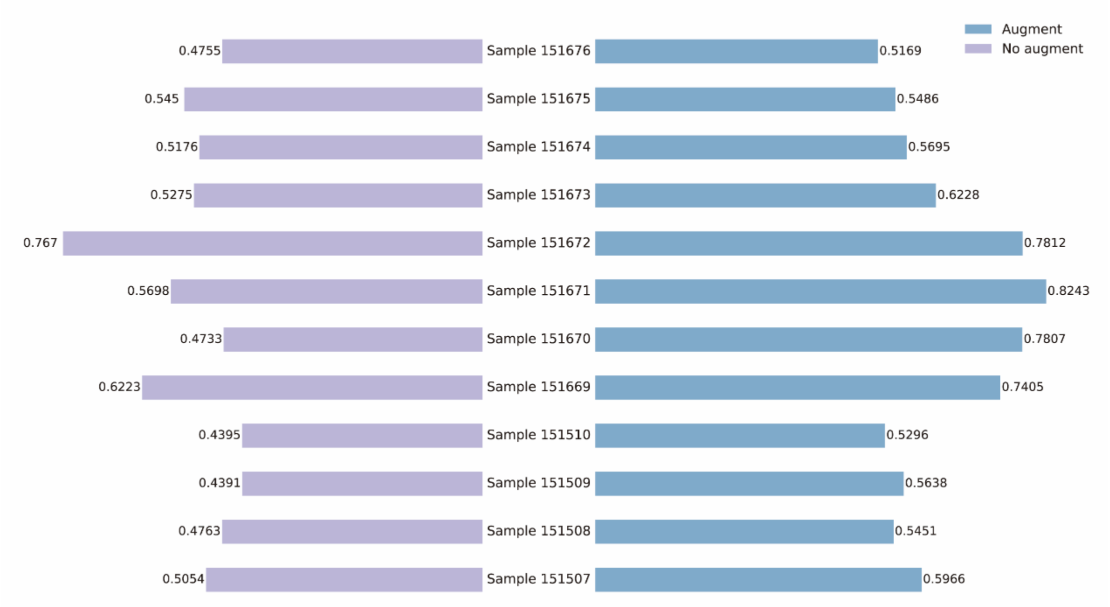

Supplementary Fig. S7. Visualization results of the effect of gene expression data augmentation on the identification of spatial domains. The purple part on the left shows the accuracy of PearlST's spatial domain identification on the 12 DLPFC samples without data augmentation, where the values indicate the ARI score. In contrast, the blue portion on the right indicates the region segmentation accuracy after data augmentation.

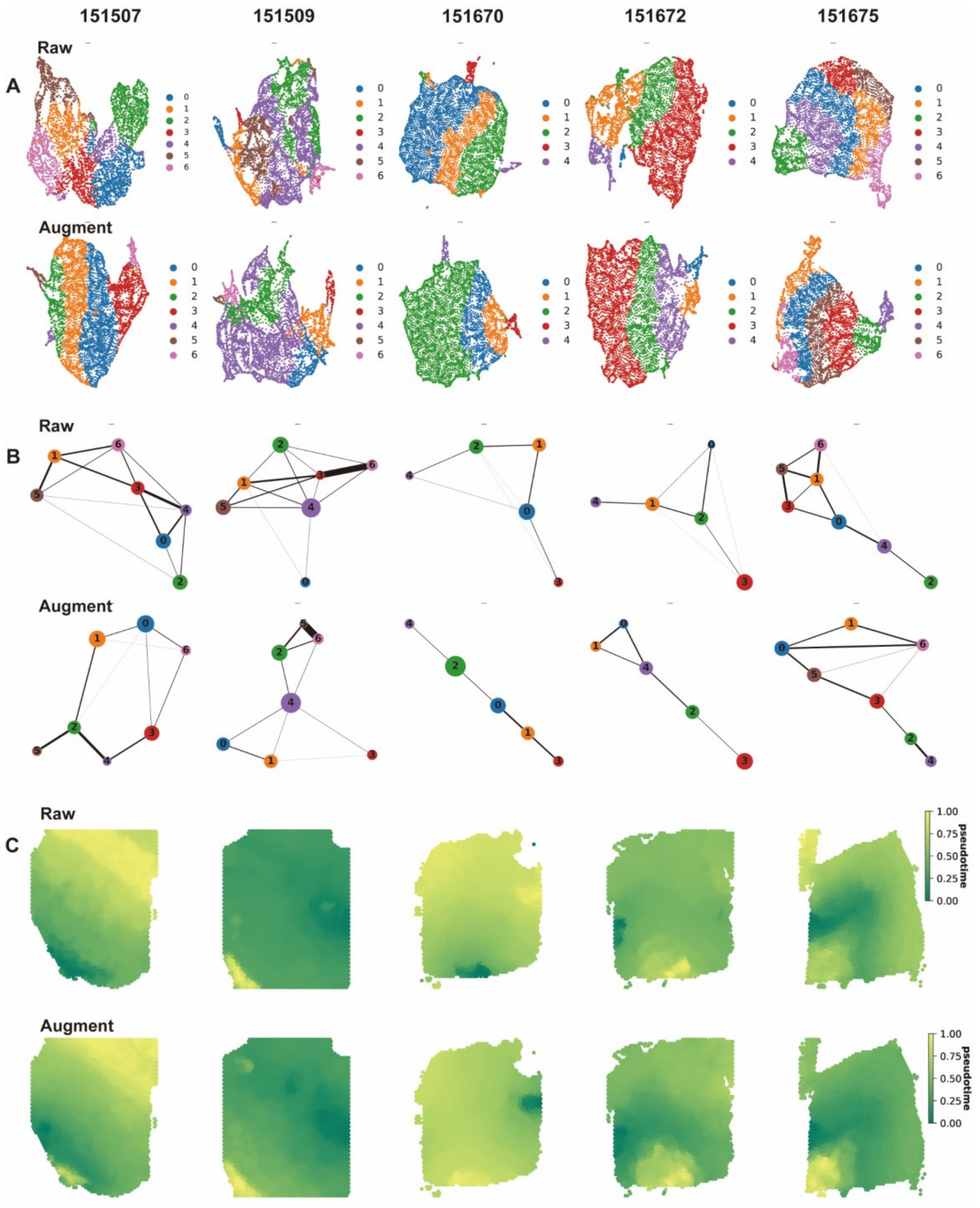

Supplementary Fig. S8. Visualization of the impact of data enhancement or not on other spatial-temporal structure inference tasks. (A) Low-dimensional visualization of comparative results on five datasets 151507, 151509, 151670, 151672 and 151675. **(B)** PAGA trajectory inference comparison results. **(C)** The comparison results of pseudo spatial-temporal map computing.

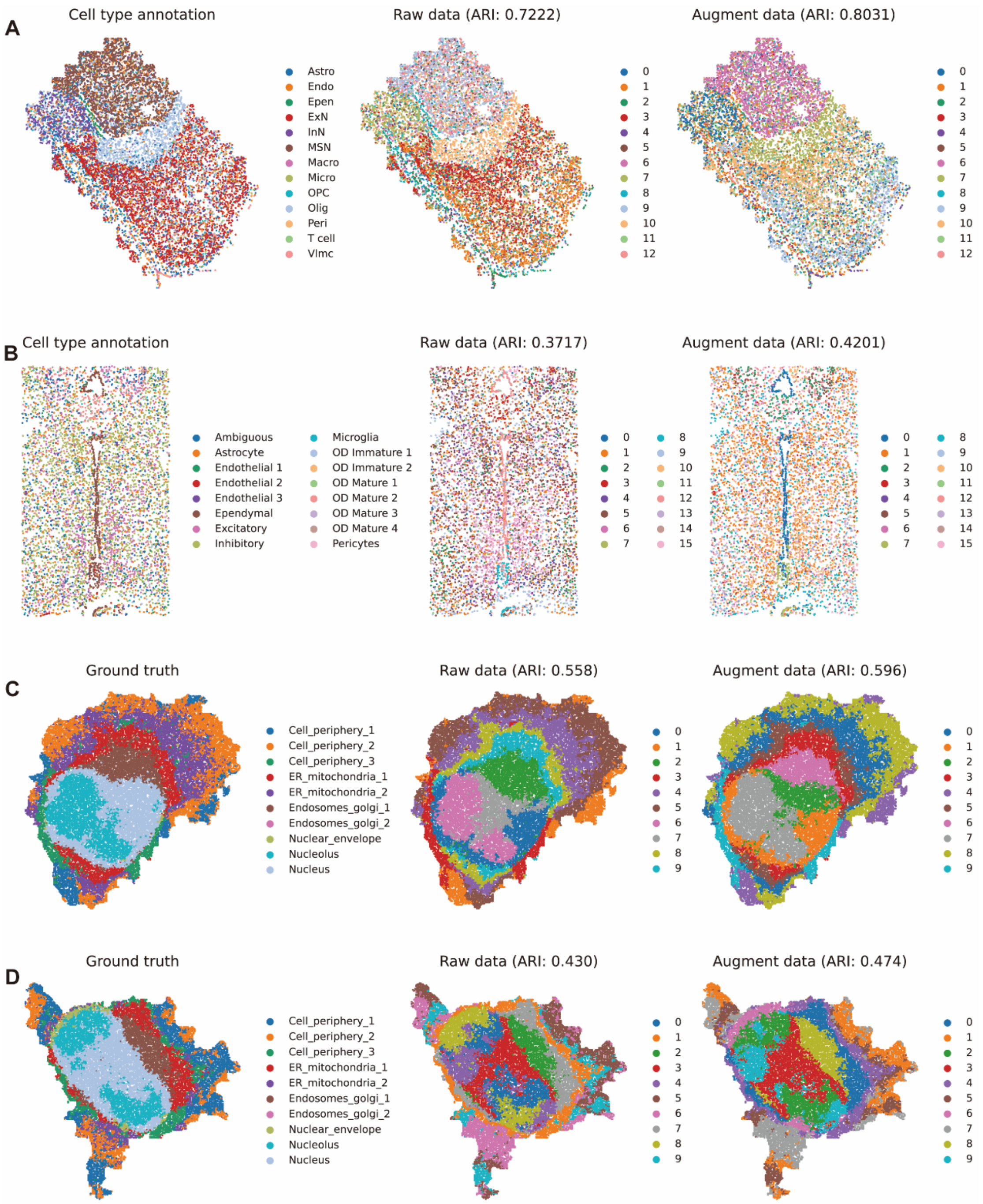

Supplementary Fig. S9. Gene expression augmentation using PDE-based diffusion model improves clustering performance on MERFISH and 4i (iterative indirect immunofluorescence imaging) data. (A) Allen2022Molecular generated by MERFISH annotation, clustering results on raw data, clustering results on augmented data. **(B)** Moffitt2018Molecular from MERFISH have been annotated into 16 cell types, this is followed in turn by the clustering results of the K-means on the raw data and augmented data. **(C)** 4i_119's ground truth, visualization of clustering results on raw data and augmented data, respectively. **(D)** Visualization of 4i_122 on manual annotations, K-means results on raw data and enhanced data.

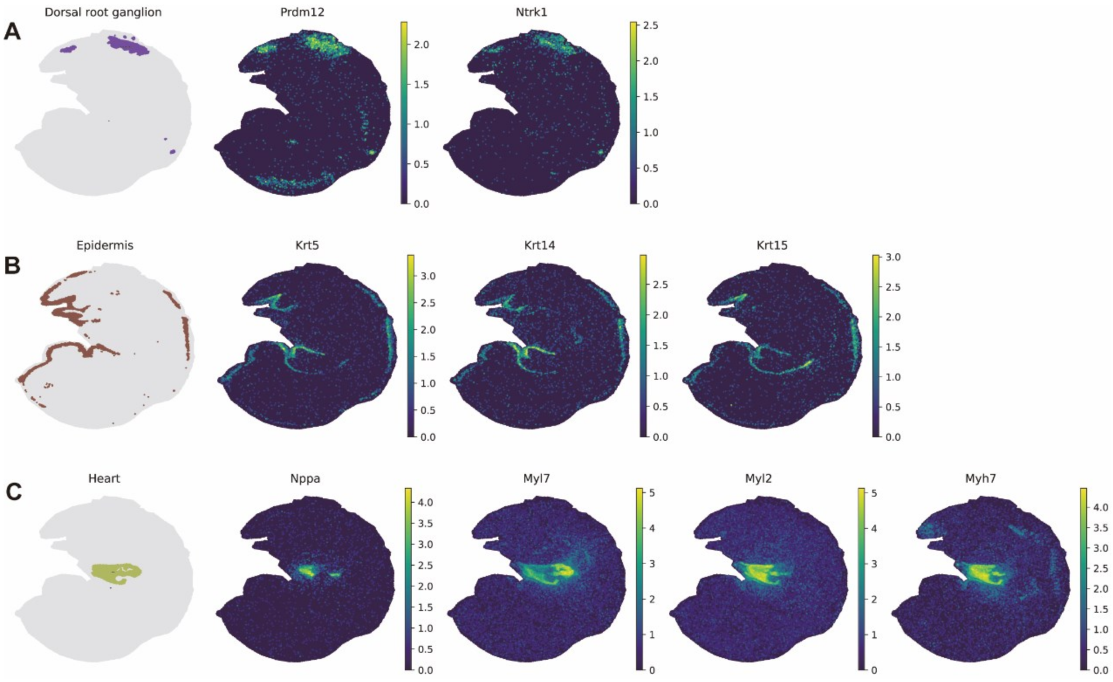

Supplementary Fig. S10. Marker genes visualization of Dorsal root ganglion, Epidermis and Heart. (A) region Dorsal root ganglion marked by Prdm12 and Ntrk1. **(B)** region Epidermis marked by Krt5, Krt14 and Krt15. **(C)** region Heart marked by Nppa, Myl7, Myl2 and Myh7.

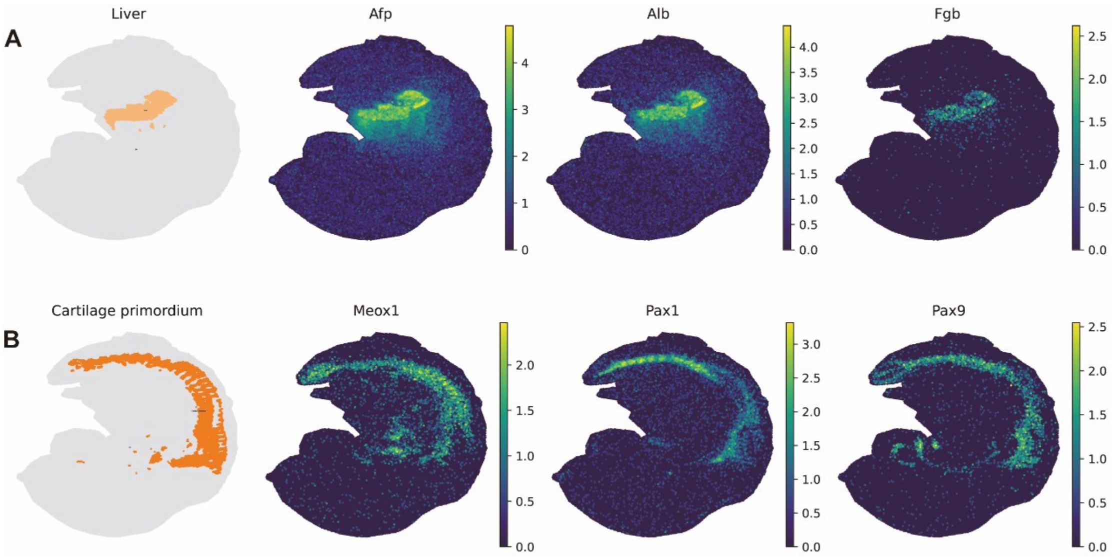

Supplementary Fig. S11. Marker genes visualization of Liver and Cartilage primordium. **(A)** domain Liver marked by Afp, Alb and Fgb. **(B)** domain Cartilage primordium marked by Meox1, Pax1 and Pax9.

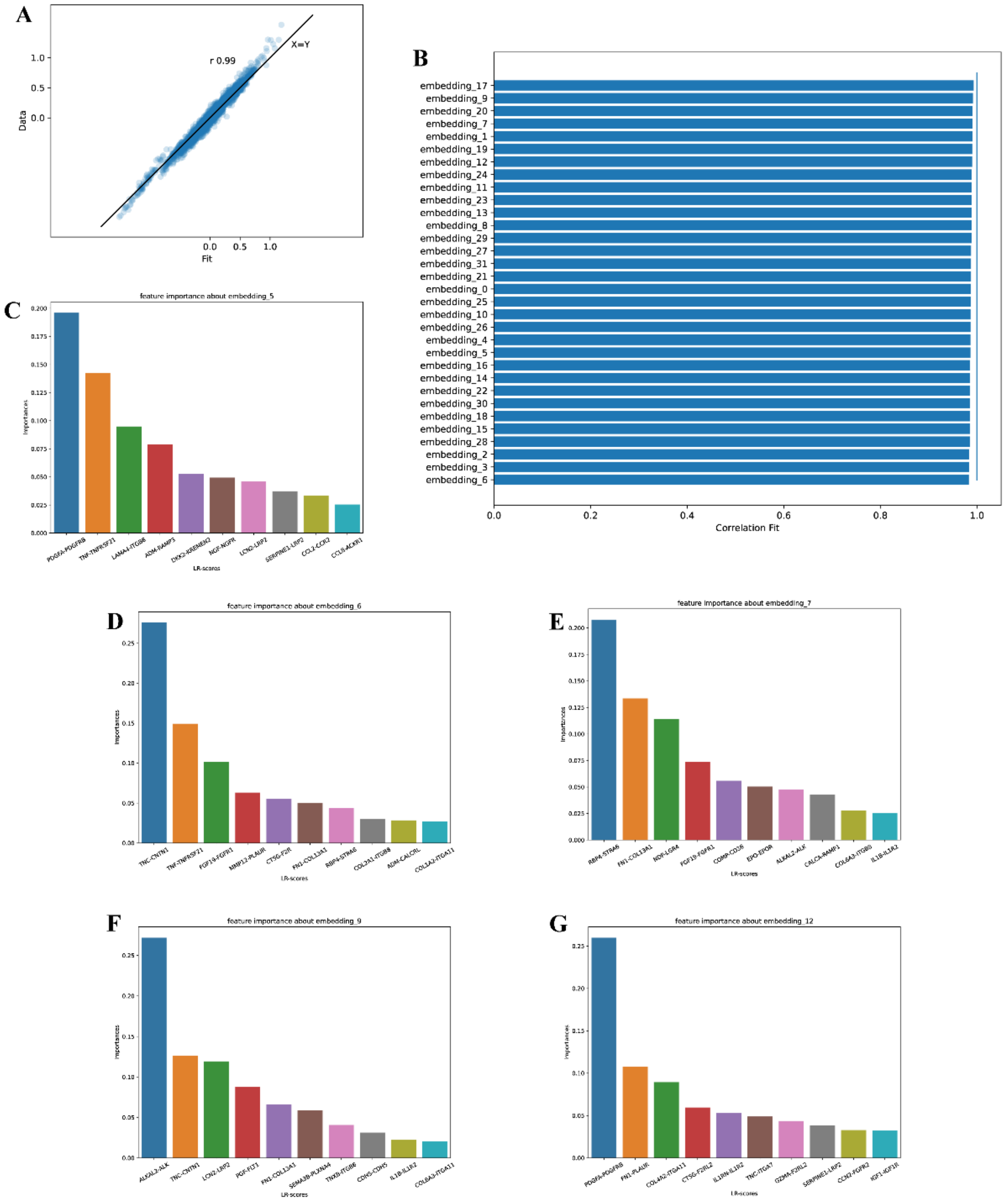

Supplementary Fig. S12. Random Forest regression characterizes the relationship between ligand-receptor scores and low-dimensional representations. (A) The fitting relationship is visualized, where r denotes the fitting coefficient, X represents the ligand-receptor scores, and Y refers the low-dimensional embeddings of the spots. **(B)** Similarity between the low-dimensional embeddings output by the random forest regression and the low-dimensional embeddings output by the original graph autoencoder. **(C)** Those ligand-receptor scores with strong regulatory effects on low-dimensional embedded_5, and the specific strength of regulatory effects. **(D)** Those ligand-receptor scores with strong regulatory effects on low-dimensional embedded_6. **(E)** The ligand-receptor scores have significant regulatory effects on low-dimensional embedded_7. **(F)** The ligand-receptor scores have dominant regulatory effects on low-dimensional embedded_9. **(G)** The ligand-receptor scores with strong regulatory effects on low-dimensional embedded_12.

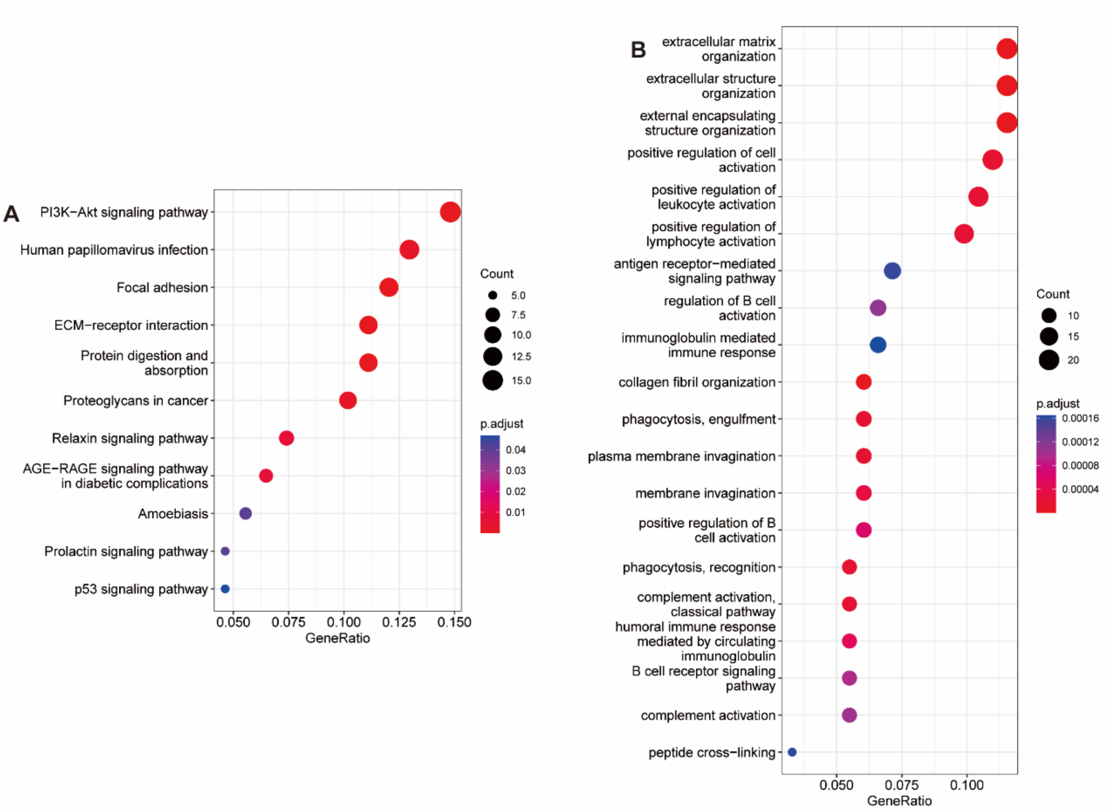

Supplementary Fig. S13. KEGG and GO gene enrichment analysis results of target genes on human breast cancer. (A) KEGG pathway enrichment results via clusterProfiler [3]. **(B)** GO enrichment results calculated by clusterProfiler.

Supplementary Reference

[1] Perona, P., & Malik, J. (1990). Scale-space and edge detection using anisotropic diffusion. IEEE Transactions on pattern analysis and machine intelligence, 12(7), 629-639.

[2] Cheng, J., Yan, L., Nie, Q., & Sun, X. (2022). Modeling and inference of spatial intercellular communications and multilayer signaling regulations using stMLnet. bioRxiv, 2022-06.

[3] Chen, K. H., Boettiger, A. N., Moffitt, J. R., Wang, S., & Zhuang, X. (2015). Spatially resolved, highly multiplexed RNA profiling in single cells. Science, 348(6233), aaa6090.

[4] Moffitt, J. R., Bambah-Mukku, D., Eichhorn, S. W., Vaughn, E., Shekhar, K., Perez, J. D., ... & Zhuang, X. (2018). Molecular, spatial, and functional single-cell profiling of the hypothalamic preoptic region. Science, 362(6416), eaau5324.

[5] Gut, G., Herrmann, M. D., & Pelkmans, L. (2018). Multiplexed protein maps link subcellular organization to cellular states. Science, 361(6401), eaar7042.
[6] Yuan, Z., Pan, W., Zhao, X., Zhao, F., Xu, Z., Li, X., ... & Yao, J. (2023). SODB facilitates comprehensive exploration of spatial omics data. Nature Methods, 20(3), 387-399.
[7] Allen, W. E., Blosser, T. R., Sullivan, Z. A., Dulac, C., & Zhuang, X. (2023). Molecular and spatial signatures of mouse brain aging at single-cell resolution. Cell, 186(1), 194-208.
[8] Yu, G., Wang, L. G., Han, Y., & He, Q. Y. (2012). clusterProfiler: an R package for comparing biological themes among gene clusters. Omics: a journal of integrative biology, 16(5), 284-287.
